# The impact of insecticide decay on the rate of insecticide resistance evolution for monotherapies and mixtures

**DOI:** 10.1186/s12936-024-05147-y

**Published:** 2025-02-18

**Authors:** Neil Philip Hobbs, Ian Hastings

**Affiliations:** 1 Department of Vector Biology, Liverpool School of Tropical Medicine, Liverpool, UK; 2 Department of Tropical Disease Biology, Liverpool School of Tropical Medicine, Liverpool, UK; 3 Present Address: Department of Epidemiology and Public Health, Swiss Tropical and Public Health Institute, Allschwil, Switzerland; 4 Present Address: University of Basel, Basel, Switzerland

## Abstract

**Background:**

The problem of insecticide decay following their deployment in public health applications is frequently highlighted as an issue for sustained disease control. There are additional concerns that it also increases selection for insecticide resistance. Despite these concerns insecticide decay is largely absent from models evaluating insecticide resistance management strategies.

**Methodology:**

The impact of insecticide decay is investigated using a model which assumes a polygenic basis of insecticide resistance. Single generation evaluations are conducted that cover the insecticide efficacy and insecticide resistance space for insecticides when deployed as monotherapies or mixtures to mechanistically investigate how insecticide decay impacts selection for resistance. The outcome is the between generation change in bioassay survival to the insecticides. The monotherapy sequence and mixture strategies were compared in multi-generation simulations incorporating insecticide decay, with the outcome being the difference in strategy lifespan.

**Results:**

The results demonstrate that as insecticides decay, they can apply a much greater selection pressure than that imposed by newly deployed, non-decayed insecticides; this applies to both monotherapies and mixtures. For mixtures, selection for resistance is highest when both insecticides have reduced decayed efficacies; this also occurs if reduced dosages are deliberately used in mixtures. Insecticide decay was found to reduce the benefit of mixtures compared to sequential monotherapies, especially when reduced-dose mixtures are used.

**Conclusions:**

Insecticide decay is often highlighted as an important consideration for mixtures and these results indicate its absence in previous modelling studies may be over-inflating the performance of full-dose mixtures. In summary: as insecticides decay, they can impose increasing selection pressures for resistance with reduced ability to control the target insect populations. The optimal frequency with which decaying insecticides should be replenished is an important policy consideration.

**Supplementary Information:**

The online version contains supplementary material available at 10.1186/s12936-024-05147-y.

## Background

Insecticides used for controlling malaria and other vector borne diseases often have long residual lifespans, i.e., they maintain their ability to kill insects for months after their initial application. Long-lasting insecticidal nets (LLINs) are expected to have an effective longevity of three years. Indoor residual sprays (IRS) are expected to have a lifespan between 3 and 9 months [1]. In reality, the practical lifespan of standard (pyrethroid-only) LLINs is rarely longer than 2 years [2–4], and this is similarly seen for dual active ingredient nets [5, 6] due to a combination of insecticide decay and physical degradation. Insecticides used as IRS also vary in longevity depending on the type of sprayed surface [7–10]. Insecticide decay is a concern for vector control because as insecticides decay over the months and years post-deployment they become less effective at killing the target vector population and, therefore, have a lower impact on controlling disease transmission. Insecticide decay is frequently highlighted as an issue for disease control programmes and there are concerns insecticide decay may undermine insecticide resistance management (IRM) strategies [11].

Important questions remain about how insecticide decay influences the dynamics of resistance evolution and subsequently the effectiveness of IRM strategies. The implication of insecticide decay for IRM was investigated by South et al*.* [12] using a single gene argument and monotherapy deployments. Their argument was that insecticide decay over time created time points where the heterozygote becomes advantageous creating “windows of selection” and “windows of dominance” during a deployment. Insecticide decay has been highlighted as a particular concern for insecticides deployed as mixtures [13]. A mixture may be highly effective when first deployed, but become counter-productive (from an IRM standpoint) as effectiveness of the constituent insecticides starts to decline. Next-generation mixture LLINs have outperformed standard (pyrethroid-only) LLINs in large cluster randomized trials [14, 15], leading to the World Health Organization (WHO) recommending chlorfenapyr-pyrethroid mixture nets over pyrethroid-only nets in areas with insecticide (namely pyrethroid) resistance as of 2023 [16]. The large-scale deployment of next-generation nets is now an operational reality. The issue of insecticide decay for mixtures on the evolution of resistance needs to be addressed as a matter of some urgency.

Computational models are frequently used for evaluating IRM strategies in both agriculture and public health [17]. Parameters describing insecticide decay are generally absent from these models [12], especially for the evaluation of strategies in a public health context. A systematic review of the resistance management literature [18] highlighted that when two insecticides are deployed in mixture they should have similar time profiles of residual efficacies. Notably, neither of the studies cited in support of this statement explicitly investigated this [19, 20]. Instead, the requirement for insecticides in mixture to have similar decay rates is presented as an intuitive argument.

In recent years there has been a resurgence in studies simulating the comparative performances of IRM strategies in public health (e.g., [21–23]). These studies have used increase computer power to cover a wider parameter space than was possible in previous studies from the 1980s (e.g., [13, 24, 25]). The larger numbers of simulations performed in these studies require more complex analysis and interpretation. Consequently, there has been a series of parallel papers designed to help explain the fundamental aspects of the results from the modelling studies, reducing the complexity of these models to better communicate the fundamentals of the results to non-specialists [26–28]. One aspect of IRM modelling which would further benefit from a detailed mechanistic explanation, is the role of insecticide decay and insecticide dose in driving resistance. The impact of insecticide dose remains controversial as it remains unclear whether increasing or decreasing insecticide doses increases or decreases insecticide resistance selection, with models disagreeing whether half- or full-dose mixtures are preferable [21, 22, 29]. Arguments around insecticide dose, particularly in agriculture, are often made on ecological principles [30], i.e., that a reduction in insecticide dose may reduce impact on non-target species but not significantly impact the level of control. However, this ecological argument does not consider the impact of reduced dose on the evolutionary process of insecticide resistance.

The previous studies have invariably used models that assume a single-gene basis of resistance. The previous work is extended by using a model which assumes a polygenic basis for insecticide resistance [31] and investigate the implication of insecticide decay on selection for resistance for both monotherapy and mixture deployments. The model developed in [31] has been used to assess various IRM strategies in scenario-specific evaluations [32–34] i.e., for mixtures, micro-mosaics and combinations.

The aim of this paper is twofold. Firstly, to provide a mechanistic explanation of how insecticide decay impacts the performance of IRM strategies, using both single and multi-generation simulations. The single-generation simulations act as informative “snap-shots” within longer-simulations, and therefore allow a better mechanistic understanding of the selection process as insecticides decay. Secondly, to understand how the inclusion of insecticide decay influences the comparative performance of IRM strategies. For this, multi-generational simulations are used to directly compare between IRM strategies when insecticides are allowed to decay during deployment.

## Methods

### Model overview

A previously described dynamic quantitative genetics model [31] is used, which assumes insecticide resistance is a polygenic trait. A key headline summary of the model methodology is given below, with the key technical details in Supplement 2. Readers are pointed to [31] for the full technical details of the model.

In the model, the level of insecticide resistance is quantified by the “polygenic resistance score” (PRS) which can be measured in standardized bioassays, such as WHO cylinder bioassays, to give an operationally interpretable measurement. The PRS is considered to be a classically polygenic trait. Insecticide selection can be implemented by either a truncating or probabilistic selection process. In truncation selection only the most resistance individuals survive selection, for example if 5% of individuals in the population are expected to survive these would be individuals with the top 5% PRS in the population. While in probabilistic selection individual survival is dependent on the individual PRS. In this project the truncation selection branch of the model (“polytruncate”) is used as it generates a better mechanistic understanding of the selection process with respect to insecticide decay. Providing these mechanistic explanations of insecticide selection makes the model more interpretable.

The key equation for the interpretation the work presented below is a modified sex-specific Breeder’s equation (Eq. 3c in [31]):

1 $$Response\ to\ Selection=Heritability*\left(\frac{(Female\ Selection\ Differential+Male\ Selection\ Differential)}{2}\right)*Exposure\ Scaling\ Factor$$

**Fig. 1:**
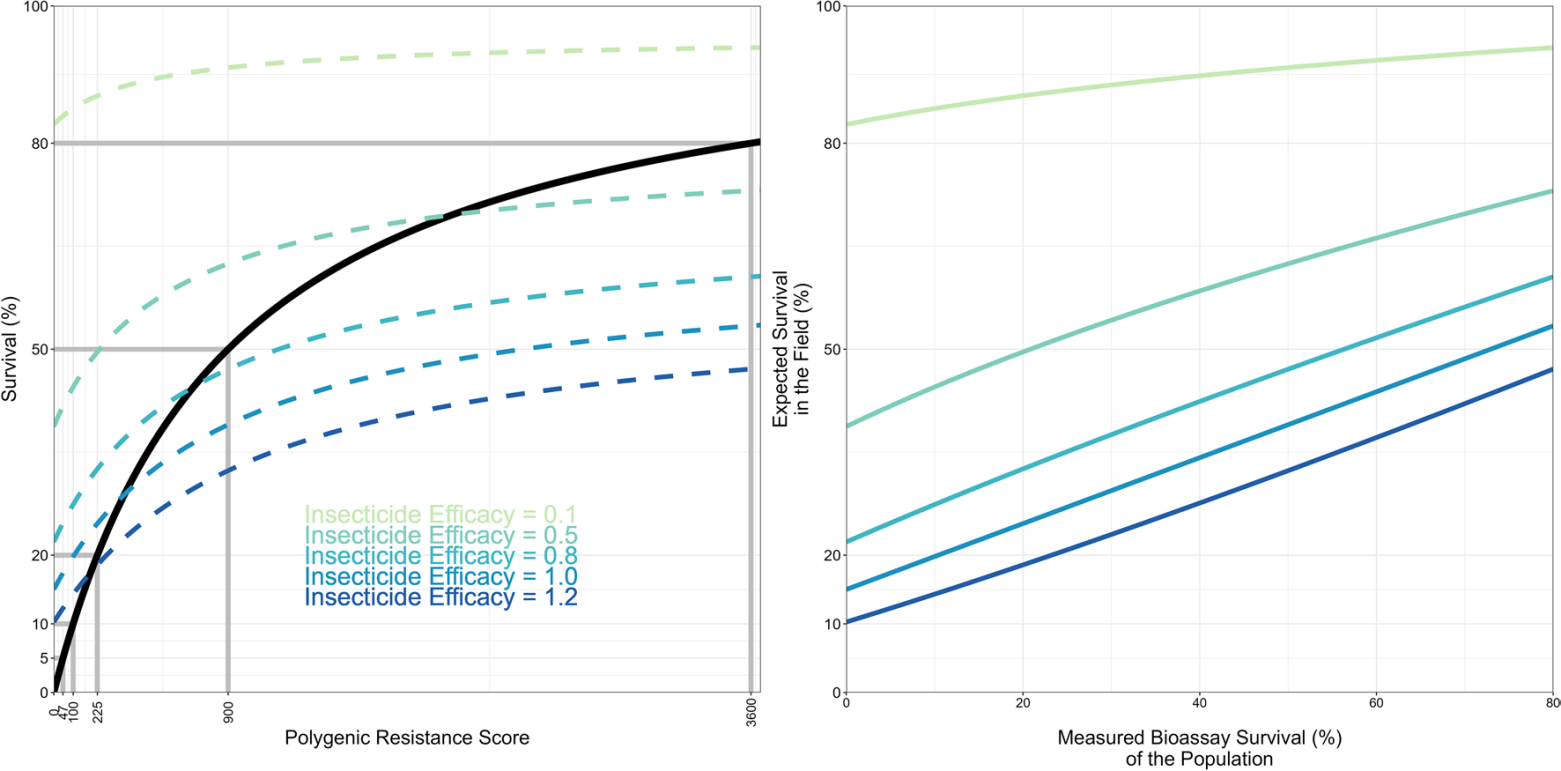
Converting the polygenic resistance score to survival. Left Panel: The solid black line is the direct conversion of the polygenic resistance score (PRS) to survival, as measured in standardized bioassays such as the WHO tube test. The survival measured in a bioassay is further converted to average survival in the field, which must account for the variation in exposure times and the efficacy of the insecticide. For details see Eqs. 1a in [21] and 2b(i) [31] for technical details. Right Panel: Conversion of the measured bioassay survival of the population, and the expected field survival to the insecticide given different insecticide efficacies. Note 1: both panels use the same colour scheme for the insecticide efficacy. Note 2: The line for the insecticide efficacy = 1 (right panel) is the identical relationship as used in [21]

**Fig. 2:**
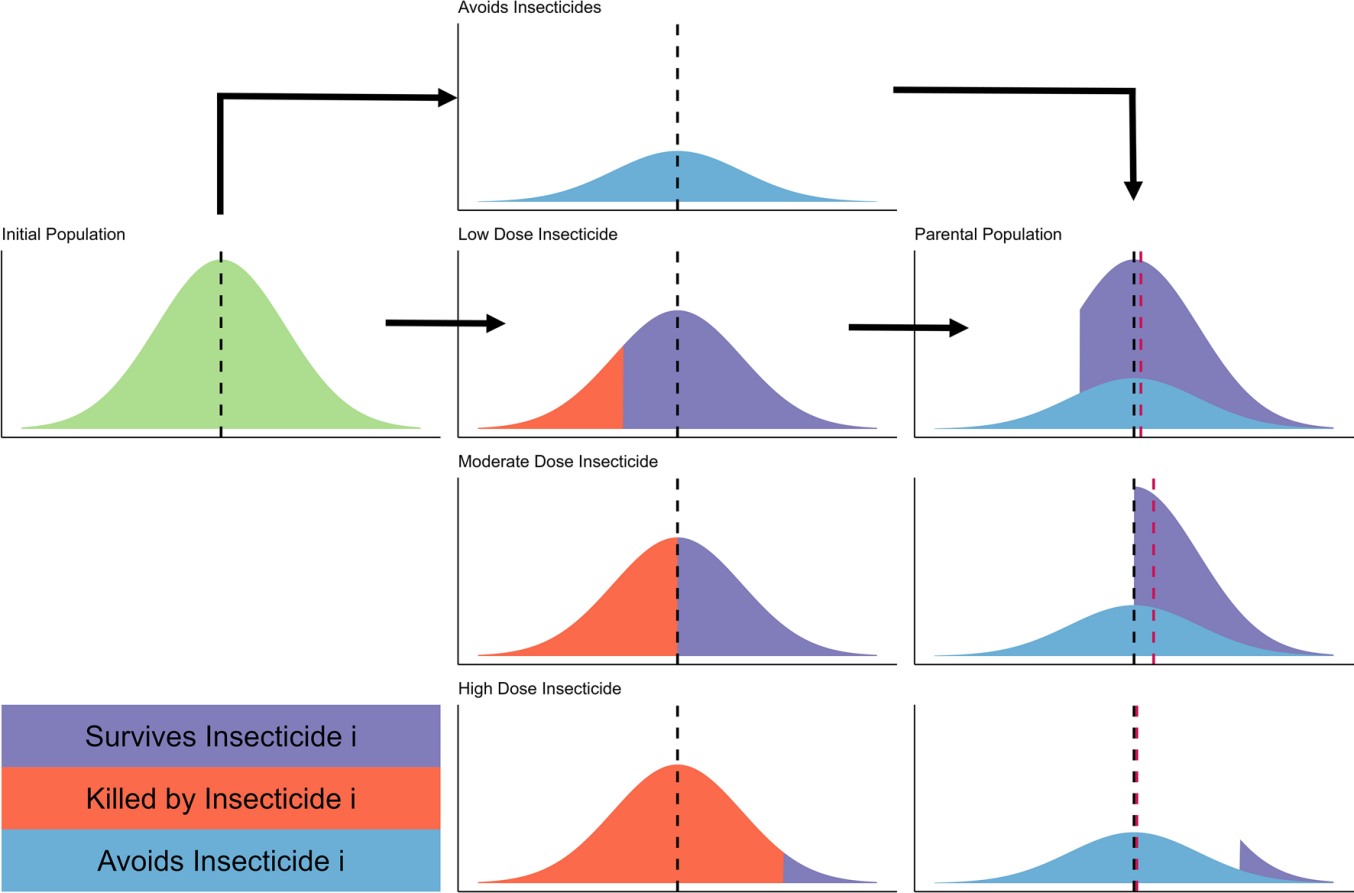
Mechanistic explanation of the impact of insecticide dose on the rate of selection for monotherapies. For all plots the x-axis is the value of the “polygenic resistance score” (PRS) and the y-axis is its frequency distribution in the population. The mosquito population initially emerges with a mean PRS (vertical black dashed line). A proportion of these mosquitoes will avoid the insecticide(s) and have no selection pressure applied to them. The other proportion of the population will be exposed to the insecticide. The number surviving insecticide contact will depend on both the dose of insecticide they encounter (because higher insecticide doses kill more mosquitoes), and the level of resistance in the population (because higher resistance populations can better survive insecticides). The selection differential, used in the Breeder’s Equation, is the change in the mean PRS between the initial emerging population (black dashed line) and the final parental population (vertical red dashed line). In scenarios where most mosquitoes survive the insecticide, (e.g., the low dose example) the selection differential is low because many of the less resistant mosquitoes survive and is further diluted by those mosquitoes escaping insecticide exposure. In scenarios where few mosquitoes survive the insecticide, (e.g., the high dose example) the selection differential is also low. This is because while those which survive the insecticide exposure are highly resistant, there are very few of these individuals. The unexposed individuals therefore make up the majority of the final parental population diluting the highly resistant survivors. In the intermediary scenarios (e.g., the moderate dose example), the insecticide imposes a selection pressure that allows a large number of moderately resistant mosquitoes to survive. The number of mosquitoes surviving the insecticide is sufficiently large such that the parental population is not sufficiently diluted by mosquitoes which avoided the insecticide

The response to selection is the between generation change in the mean PRS of the population, that is the change in the *genetic* level PRS between the parents of a generation and their offspring which survive to become parents of the next generation. The selection differentials are the within generation change in the mean PRS. Heritability is the proportion of the phenotypic variation that is genetic. The Breeder’s equation is applied separately for resistance selection by each insecticide and the corresponding resistance trait. Male and female mosquitoes may have different exposures to insecticides so the selection differentials are calculated separately for each sex. The exposure scaling factor is used to calibrate simulations to expected timescales to account for uncertainty in values of heritability and the selection differentials [21]. Calculations of the selection differentials and responses are conducted using the PRS scale, but are reported as changes in bioassay survival, where the PRS is converted to bioassay survival using a Hill-variant of the Michaelis–Menten equation (see Eq. 1a in [21] for details). This is because operational decision-making is likely to use measurements of bioassay survival to inform insecticide deployments. A graphical example of the conversion of the PRS to bioassay survival calculations is presented in Fig. 1. This figure also includes the conversion for accounting for field exposure to insecticides and insecticide efficacy, using Eq. 2b(i) in [31]. Figure 2 illustrates a simplistic mechanistic example of how insecticide deployment determines the selection differential for monotherapy deployments.

The model is used to explore insecticide decay in two ways. First, selection is calculated over a single generation as this allows a detailed understanding of the direct impact of insecticide decay on selection for resistance, allowing a detailed mechanistic understanding of the underlying selection process resulting from insecticide decay for monotherapies (mechanistically explained in Fig. 2) and mixtures (mechanistically explained in Fig. 4). Second, multi-generational scenarios of IRM strategy deployments are used to explore how including insecticide decay over longer timescales impacts the comparative ability of monotherapies and mixtures to delay the spread of IR over a 50-year time horizon.

**Fig. 3:**
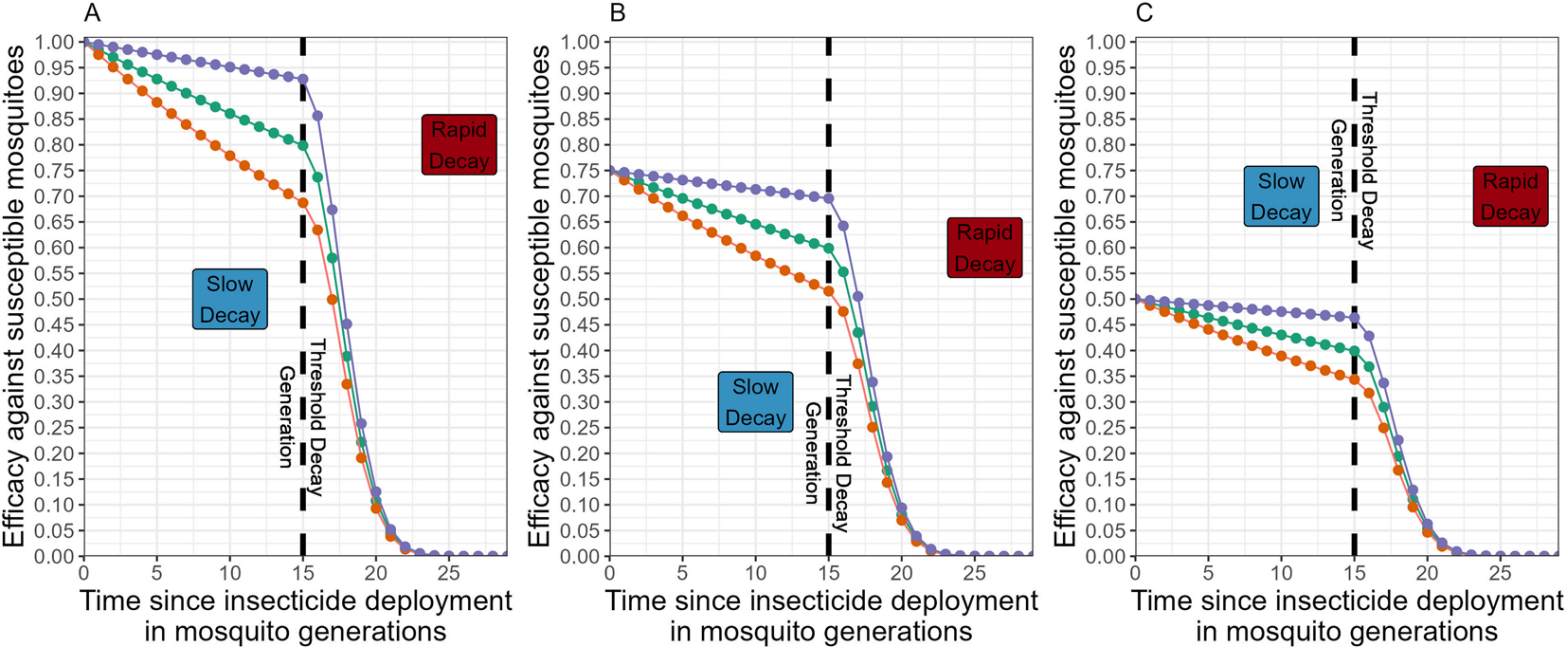
Example insecticide decay profiles for LLINs assuming a two-stage decay. After insecticides are deployed, their efficacy will decrease over time (i.e., “decay,”). Insecticide decay rates are likely a function of insecticide chemistry and fabric integrity, resulting in two-stage dynamics of insecticide decay. The colour of the lines indicates the base decay rate of the insecticide which occurs during the first 15 generations (1.5 years) of deployment and is 0.005 for purple, 0.015 for green (estimated default) and 0.025 for orange, this is the period during which there is slower decay. After 15 generations (1.5 years) (estimated default) the insecticide efficacy starts to decay rapidly, and the decay rate for this period is 0.08 (estimated default). Panel **A** is when the insecticide is deployed at the recommended dose, as would occur with monotherapy deployments and full-dose mixtures. Panel **B** and **C** are when the insecticide is deployed at a reduced dose as may occur with mixture deployments, and the reduced dose results in the initial efficacy being reduced to either 0.75 (Panel **B**) or 0.5 (Panel **C**). Details of parameter estimation are in Supplement 1

### Insecticide decay and monotherapy deployments: changes in insecticide resistance and control over a single generation

First single-generation simulations are used to explore and understand the insecticide resistance selection process. These single-generation simulations act as snap-shots within longer multi-generational simulations providing a mechanistic explanation of how as insecticides decay in efficacy this impacts the rate of insecticide resistance selection.

Insecticide decay when only one insecticide is deployed (monotherapy) is first considered. The parameter ${\omega }_{\tau }^{i}$ is the killing efficacy of insecticide $i$ against fully susceptible mosquitoes (i.e. those with PRS = 0), $\tau$ generations after deployment in standardized bioassays (e.g., WHO tube tests). The value ${\omega }_{\tau }^{i}$=1 indicates the insecticide is at the recommended dose and kills 100% of susceptible mosquitoes (${z}_{I}$ ≤ 0) in a bioassay. This would be analogous to the LD_100_ for a fully susceptible mosquito population. Values where ${\omega }_{\tau }^{i}$>1 indicate the insecticide is above the recommended dose (i.e., the dose is above the LD_100_ for a fully susceptible mosquito population), as may occur with over-spraying for IRS or when considering increasing the dose to reduce the threat of resistance. Insecticide efficacy in the model is defined as the ability of the insecticide to kill fully susceptible mosquitoes in a bioassay (PRS $\le 0$), such that an insecticide efficacy of 1 kills all susceptible mosquitoes in standardized bioassays [31]. Bioassay survival is converted to field survival (Fig. 1, right panel), so as to account for variable durations of insecticide contact by mosquitoes for example.

As insecticides decay there is a reduction in efficacy (${\omega }_{\tau }^{i}$) which is calculated using Eqs. 2d(i) and 2d(ii) in [31] and illustrated in Fig. 2 presented here. Details of insecticide decay rates estimation are in Supplement 1. Single generation selection (Fig. 2) was conducted across a range of insecticide efficacy (${\omega }_{\tau }^{i}$) with values from 0 to 1.2 at intervals of 0.1. Single generation selection does not account for how long insecticides remain at these efficacies which would be dependent on the specific decay profile (Fig. 3) but provides a mechanistic understanding of the selection process and the implication of insecticide decay.

**Fig. 4:**
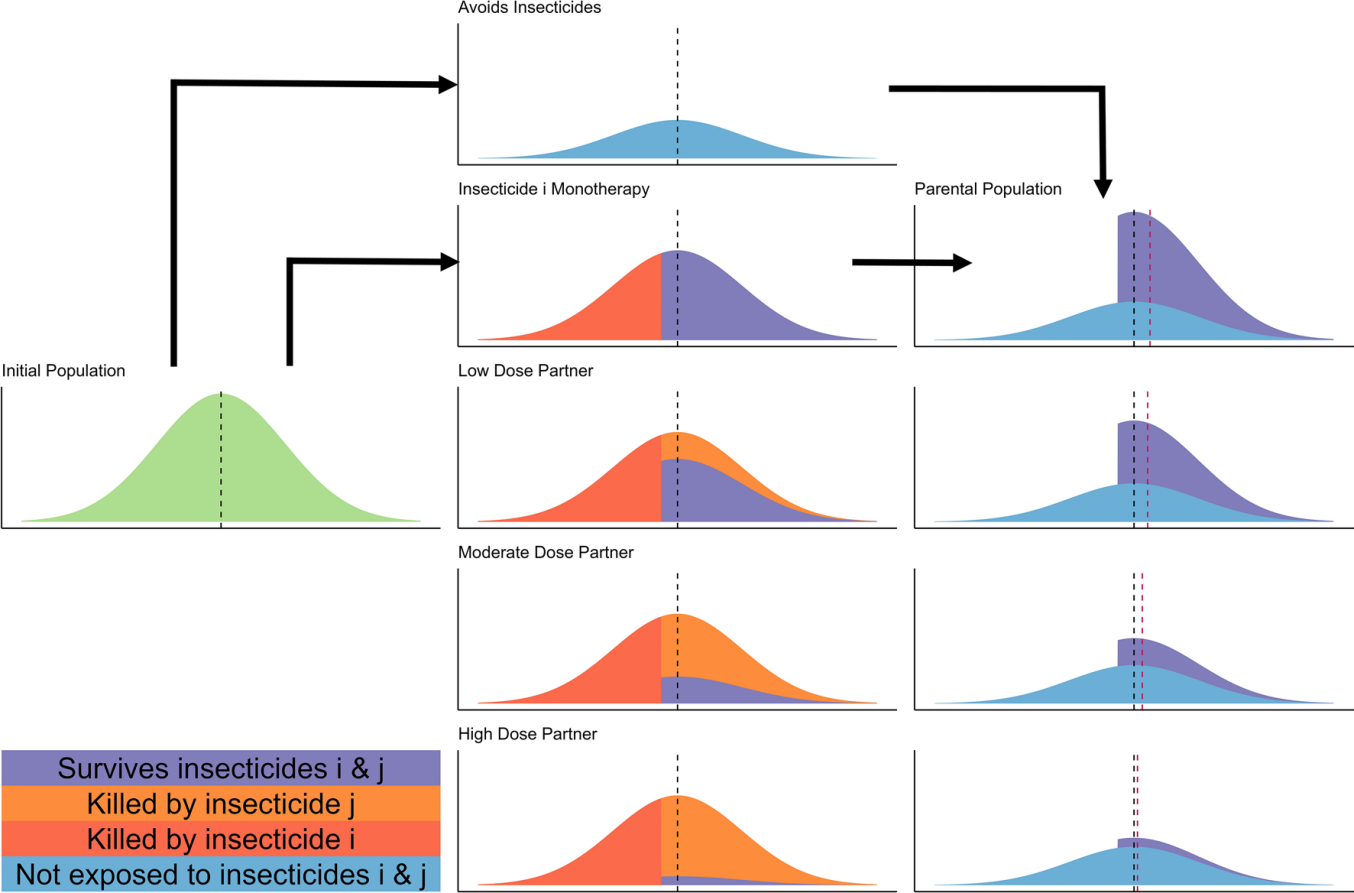
Mechanistic explanation of the impact of insecticide dose on the rate of selection: mixtures. For all plots the x axis is the value of the “polygenic resistance score” (${z}_{I}$) and the y axis is the frequency in the population. The initial mosquito population emerges with a mean resistance of ${\overline{z} }_{I}$ to insecticide $i$ and a mean resistance of ${\overline{z} }_{J}$ to insecticide $j$. A proportion of these mosquitoes will avoid the insecticide(s) and have no selection pressure applied to them. The other proportion of the population will be exposed to the insecticide(s). The number surviving will depend on the dose of insecticide (higher insecticide doses kill more mosquitoes), and the level of resistance in the population (higher resistance populations can better survive insecticides). For scenarios where mosquitoes are exposed to a mixture insecticide $i$ kills a proportion of those mosquitoes, depending on the level of resistance to insecticide $i$ and the dosage of insecticide $i$ (red). Those which survive insecticide $i$ then also must survive insecticide $j$ (which depends on the level of resistance to insecticide $j$ and the dosage of insecticide $j$), and therefore insecticide $j$ kills an additional proportion of the mosquitoes (orange). To become parents of the next generation, mosquitoes most therefore survive both insecticides (purple). This conceptual framework can be used to explain how mixtures affect selection for resistance. If the partner insecticide ($j$) is not effective (low dose and/or high levels of resistance) then only a small additional number of mosquitoes are killed, only slightly reducing the selection differential compared to insecticide $i$ being deployed in monotherapy. If the partner insecticide ($j$) is effective (high dose and/or low levels of resistance) then insecticide $j$ kills a large number of mosquitoes, which were more resistant to insecticide $i$. The selection differential (within generation change in mean resistance) is therefore the difference in the initial mean (dashed black line, ${\overline{z} }_{I}$) and the mean of the parents (red dashed line, ${\overline{z} }_{I}^{P}$)

The impact of insecticide decay on the response to selection was then further assessed across a range of resistance and exposure values. The initial resistance of the population (as measured bioassay survival, ${K}_{i}^{B}$) was set as 0, 5, 10, 20, 50 or 80% (Table 1). These populations have differing exposure rates (0.1 to 1 at 0.1 intervals), with male and female mosquitoes having the same exposure rates. Exposure is defined as the proportion of the mosquito population which contact the insecticide(s). Heritability (${h}_{I}^{2}$) was set at 0.2. Higher and lower heritability values increase and decrease the rate of evolution respectively (see modified sex-specific Breeder’s equation) but do not impact the interpretation of the results. In the single-generation evaluation dispersal to/from untreated an untreated refugia (i.e., areas where the insecticide(s) are not deployed) and fitness costs of resistance were not included to simplify interpretation. This is relaxed in the multi-generational scenarios later.

**Table 1:** Single generation change in bioassay survival: Monotherapy Deployments

Current Efficacy of Insecticide $i$ (${\omega }_{\tau }^{i}$) against a fully susceptible population		Mean Polygenic Resistance Score of the population to insecticide $i$	Corresponding Bioassay Survival to insecticide $i$ (${K}_{i}^{B}$) (%) of the population		Female exposure rate ($x$)		Outcomes
1.2		0	0		1	X	• Response to selection measured as change in bioassay survival for insecticide $i$ over a single mosquito generation • “Degree of Control”
1.1					0.9		
1					0.8		
0.9		47	5		0.7		
0.8					0.6		
0.7		100	10		0.5		
0.6	X			X	0.4		
0.5		225	20		0.3		
0.4							
0.3		900	50		0.2		
0.2							
0.1		3600	80		0.1		
0							

Secondly the “degree of control” provided by the insecticide at its current efficacy is calculated. This is the proportion of the adult female population which are killed within a single generation. The proportion of female mosquitoes which would be expected to survive to lay a single batch of eggs and, therefore, successfully complete a single gonotrophic cycle is calculated. This number is calculated as the number after treatment (purple and blue areas in Fig. 2) divided by the number before treatment (green area Fig. 2). A formal explanation is in Eq. 4c [31]. This is simply reported as the “degree of control”:

2 $$Degree\ of\ Control=1- \left(\frac{Population\ Size\ after\ Treatment}{Initial\ Population\ Size\ before\ Treatment}\right)$$

As the model does not link into a population dynamics model, it is important to stress that this is a simplistic single-generation interpretation. This simplification inevitably ignores the complexities around mosquito demographics (i.e., older female mosquito are more important for transmission), and the complexities around population regulation over successive generations (e.g., density dependence), and the specifics of how the insecticide is deployed (e.g., as an ITN or IRS). These calculations are performed across the ranges of efficacy, resistance, and exposure values as detailed above. The “degree of control” scale is such that when the “degree of control” = 1, then all mosquitoes are killed by insecticide. When the “degree of control” = 0, the insecticidal intervention has no effect.

All permutations of insecticide efficacy, exposure, and resistance were run (Table 1) to investigate how these three parameters interact. The primary outcome was the response to selection over a single generation as defined in the Breeder’s equation, i.e., the increase in insecticide resistance over a single mosquito generation as measured by change in the bioassay survival, with the “degree of control” being a secondary measure.

### Insecticide decay and mixture deployments: change in insecticide resistance and control over a single generation

The logical next step evaluates the impact of insecticide decay when two insecticides are deployed in a mixture. Mixtures contain two insecticides (denoted insecticides $i$ and $j$ in this manuscript) within the same formulation such that mosquitoes are inevitably exposed to both insecticides simultaneously. Single generation selection was conducted (Fig. 4) across the range of insecticide efficacy values (${\omega }_{\tau }^{i}$ and ${\omega }_{\tau }^{j}$) to again provide snap-shots into the mechanistic process occurring during multi-generation simulations. Insecticide $i$ and $j$ can have different efficacies as would occur if they had different decay profiles or if one was deployed at sub-optimal initial concentrations. Insecticide efficacy parameter values were investigated at 0.1 intervals ranging from 0–1.2 for each insecticide.

The impact of the exposure (the proportion of the mosquito population which contact the insecticide(s)) and level of resistance to the insecticides was also explored. The ranges were the same as for the monotherapy investigations i.e. (i) Exposure to the mixture was set at 0.1 to 1 at 0.1 intervals, with male and female mosquitoes having the same exposure (ii). Initial resistance to the insecticides as bioassay survival (${K}_{i}^{B}$ and ${K}_{j}^{B}$) was set as 0, 5, 10, 20, 50 or 80% for each insecticide, but for mixtures allowing the level of resistance to the two insecticides in the mixture to be different. (iii) Heritability (${h}_{I}^{2}$ and ${h}_{J}^{2}$) to each insecticide was fixed at 0.2 in all the single-generation simulations. (iv) Dispersal to/from refugia and fitness costs were not included to simplify interpretation. All permutations of these values were run (as described in Table 2). This allows us to explore the entirety of the efficacy-resistance-exposure space for mixtures providing detailed mechanistic understanding of the selection process. Similarly with the monotherapies, the “degree of control” is additionally calculated using the methodology previously described.

**Table 2:** Single generation change in bioassay survival: Mixture Deployments

Current Efficacy of Insecticide $i$ (${\omega }_{\tau }^{i}$) against a fully susceptible population		Current Efficacy of Insecticide $j$ (${\omega }_{\tau }^{j}$) against a fully susceptible population		Mean Polygenic Resistance Score of the population to insecticide $i$	Corresponding Bioassay Survival to insecticide $i$ (${K}_{i}^{B}$) (%) of the population		Mean Polygenic Resistance Score of the population to insecticide $j$	Corresponding Bioassay Survival to insecticide $j$ (${K}_{j}^{B}$) (%) of the population		Outcomes
1.2		1.2		0	0		0	0		• Single generation bioassay change for insecticide $i$ • Single generation bioassay change for insecticide $j$ • Combined total of bioassay change for insecticide $i$ plus bioassay change for insecticide $j$ as measure of the “total amount of selection” • “Degree of Control”
1.1		1.1								
1		1								
0.9		0.9		47	5		47	5		
0.8		0.8								
0.7		0.7		100	10		100	10		
0.6	X	0.6	X			X			X	
0.5		0.5		225	20		225	20		
0.4		0.4								
0.3		0.3		900	50		900	50		
0.2		0.2								
0.1		0.1		3600	80		3600	80		
0		0								

The primary outcome was the response to selection measured as the changes in bioassay survival over a single generation (see Breeder’s Equation and Fig. 4). This is measured for both insecticides separately and are added together to get a measure for the total amount of selection. The responses are plotted to visualize regions of the mixture efficacy and resistance space which give higher and lower selection on insecticide $i$, insecticide $j$ and both insecticides to gain an understanding of the implication of insecticide decay for mixtures.

### Impact of insecticide decay on IRM strategy lifespans: multi-generational changes in insecticide resistance for monotherapies and mixtures

Clearly, an important consideration is how long insecticides remain effective during a deployment, which will depend on the decay profiles of the insecticides (Fig. 3 and Fig. S3.1 in Supplement 3). Insecticide decay rates for a standard pyrethroid LLIN collected from the field over time [6] were estimated (Supplement 1) to give default parameter values for the decay rate. Many factors can influence the rate of insecticide decay in the field such as the substrate for IRS [35] and household conditions for LLINs [5]. Therefore, a set of illustrative example two-stage decay profiles was examined (Fig. 3). These decay profiles are used to assess the impact of slower or faster decay rates on the performance of IRM strategies. Simulations were also run where insecticide decay did not occur as this is the usual assumption in most models. Including simulations both with/without decay provides a direct comparison to understand whether the inclusion of insecticide decay both quantitatively changed the long term sustainability of IRM strategies (i.e. time until resistance made the insecticides ineffective), and more importantly whether the inclusion of insecticide decay changed the qualitative conclusion of which strategy performed best. To further assess how our assumption of a two-stage decay process influences the results, simulations assuming the insecticides decayed at a constant rate throughout their post-deployment life-time were additionally run as has been assumed in other studies [36] (detailed in Supplement 3).

Simulations were run to compare two insecticides deployed as monotherapies in sequence, which is generally the default IRM strategy versus the deployment of the same insecticides as a mixture. The properties of the insecticides were the same in both strategies allowing for direct comparisons. The initial dose for mixtures was set so that efficacy was 100%. The 75% and 50% efficacies were used to emulate the impact of insecticides in mixture being deployed at a reduced dose.

The model additionally allows for the inclusion of cross resistance (${\alpha }_{IJ}$) as a correlated response (Eq. 8b and 8c in [31]). The implication of cross resistance between the two insecticides is additionally explored as this is considered an important operational concern and is likely to interact with insecticide decay. Previously full-dose mixtures were found to be superior to monotherapy sequences regardless of the level of cross resistance [21, 31], however this did not consider insecticide decay. Cross resistance is now included as either positive (${\alpha }_{IJ}$ = 0.3), negative (${\alpha }_{IJ}$ = -0.3) or absent (${\alpha }_{IJ}$ = 0).

For all multi-generation simulations, unless otherwise stated, the default parameter values are those detailed in Table 3. Three separate scenarios were conducted to investigate the impact of insecticide decay. Scenario 1 assumed both insecticides started at the same resistance level and had the same heritability. This focusses on the impact of insecticide decay. Scenario 2 assumed the insecticides started at different levels of resistance. Scenario 3 assumed the insecticides had different heritabilities. Details of the parameter values used for each scenario are given in Table 3.

**Table 3:** Parameter values for simulation scenarios

Default parameter values		
Parameter	Symbol(s)	Value(s)
Coverage	$C$	0.7
Dispersal	$\theta$	0.2
Heritability	${h}_{I}^{2}$ and ${h}_{J}^{2}$	0.2
Female exposure	$x$	0.7
Male exposure	$m$	1 (Male exposure is a proportion of female exposure, setting $m$ =1 means male and female exposure are the same)
Initial mean PRS	${\overline{z} }_{I}$ and ${\overline{z} }_{J}$	0
Cross resistance	${\alpha }_{IJ}$	− 0.3, 0, 0.3
Fitness cost selection differential	${S}_{I}^{\upphi}$^♀^, ${S}_{I}^{\upphi}$^♂^, ${S}_{J}^{\upphi}$^♀^,${S}_{J}^{\upphi}$^♂^	0
Relationship between bioassay survival and field survival (regression coefficient)	${\varphi }_{1}$	0.48 [21]
Relationship between bioassay survival and field survival (regression intercept)	${\varphi }_{2}$	0.15 [21]
Standard deviation	${\sigma }_{I}$	20 [31]
Exposure Scaling Factor	$\beta$	1 [31]
Base insecticide decay rate (insecticide efficacy per generation)	${{\delta }_{\text{b}}}^{i}$	0.005, 0.015, 0.025
Threshold generation	${\tau }_{\text{b}}$	15
Rapid decay rate (insecticide efficacy per generation)	${{\delta }_{\text{r}}}^{i}$	0.08
Deployed insecticide efficacy	${\omega }_{0}^{i}$	1, 0.75, 0.5
**Scenario 1: uses the default parameters**		
**Scenario 2: mismatched initial resistance**		
Initial mean PRS (and corresponding bioassay Survival)	${\overline{z} }_{I}$ (${\overline{K} }_{i}^{B}$)	25 (2.7% Bioassay Survival)
Initial mean PRS (and corresponding bioassay Survival)	${\overline{z} }_{J}$ (${\overline{K} }_{j}^{B}$)	0 (0% Bioassay Survival)
**Scenario 3: mismatched heritabilities**		
Heritability insecticide $i$	${h}_{I}^{2}$	0.15
Heritability insecticide $j$	${h}_{J}^{2}$	0.25

Simulations were run using an insecticide withdrawal threshold of 10% bioassay survival and an insecticide return threshold of 8% bioassay survival (see Table 4 for definitions). Simulations were terminated when (a) they reached the 500-generation maximum duration with one or both insecticides still effective or (b) when no insecticides were available for deployment because resistance to both insecticides remained above the return threshold. For insecticides in mixture, if one of the insecticides reached the withdrawal threshold, the mixture was no longer available to be deployed and the simulation is terminated as the mixture strategy has “failed”. The deployment frequency was 30 generations (~ 3 years), which is the standard time between LLIN deployments. The outcome was the difference in strategy lifespan (measured in years, assuming 10 mosquito generations per year) between the mixture simulations and the comparator monotherapy sequences simulations. The use of strategy lifespan (measured as a time to a set bioassay survival or a specified resistance allele frequency) has been used previously to evaluate the performance of IRM strategies, albeit in the absence of insecticide decay. Using strategy lifespan allows for comparison with other modelling studies [21–23] which did not include insecticide decay. The model used in this study is not able to calculate the impact on mosquito population dynamics and demographics over multi-generation simulations, so how the degree of control changes over the course of these multi-generational simulations is not able to be reported.

**Table 4:** Terminology and definitions

Term	Definition
Mixture	A singe insecticide formulation which contains two insecticides, such that if a mosquito contacts the mixture they inevitably contact both insecticides
Monotherapy	The deployment of a single insecticide
Sequence	Insecticides are deployed until they reach the withdrawal threshold (see below), after which they are withdrawn from deployment and replaced with the next insecticide
Withdrawal threshold	The level of resistance (measured in bioassay survival), which leads to an insecticide being regarded as ineffective and no longer available for deployment. In this paper the withdrawal threshold is 10%
Return threshold	The level of resistance (measured as bioassay survival), which allows a previously withdrawn insecticide to be reclassified as “effective” and available for redeployment. In this paper the return threshold is 8%
Exposure	The proportion of the mosquito population which contact the insecticide(s)
Deployment frequency	The time frame between insecticide deployments

## Results

### Insecticide decay and monotherapy deployments: change in insecticide resistance level and control over a single generation

**Fig. 5:**
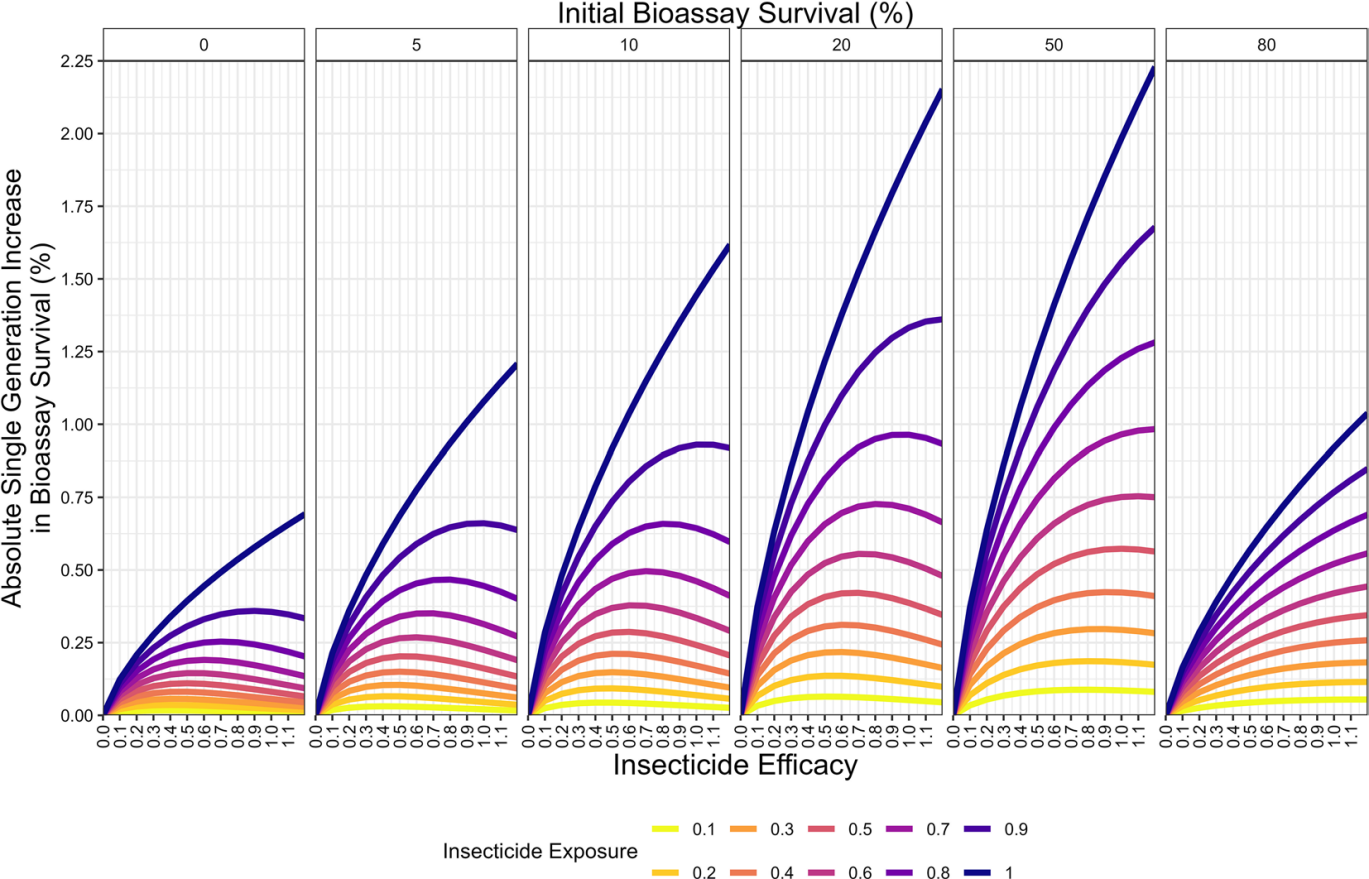
Impact of insecticide efficacy, exposure and resistance on the single generation changes in bioassay survival (%) for monotherapy deployments. Colours indicate the insecticide exposure (where the level of exposure is the same for males and females). The panels reflect different levels of initial insecticide resistance (measured as bioassay survival) present in the population prior to insecticide exposure

The results first consider the deployment of insecticides as monotherapies, such as happens with the deployment of pyrethroid-only LLINs. Figure 5 shows the impact of reductions in efficacy (e.g., due to insecticide decay) on the resulting singe-generation response to selection and how this depends on exposure rate and initial resistance level. Figure 5 shows that the rate of selection depends on the interaction between exposure, resistance, and insecticide efficacy, such that as the insecticide decays there can be higher levels of selection. These results are explained mechanistically by referring to Fig. 2.

At high insecticide efficacy (e.g., a newly deployed insecticide, Fig. 2: high dose insecticide), the insecticide kills most of the mosquitoes which are exposed to it, and the only survivors are the most resistant individuals in the population. The proportion of the population which avoided the insecticide (and therefore avoids insecticide selection) makes up most of the final parental population. The resulting response to selection is therefore small. As expected, as insecticide exposure increases then so does the response to selection (Fig. 5).

When insecticide efficacy is low (e.g., the insecticide has been deployed for a long time with much of the insecticide activity decaying away, Fig. 2: low dose insecticide) most of the mosquitoes exposed to the insecticide survive, with the insecticide killing only the most susceptible individuals. Therefore, the response to selection is small and is further reduced when the final parental population is diluted by mosquitoes which avoided the insecticide.

**Fig. 6:**
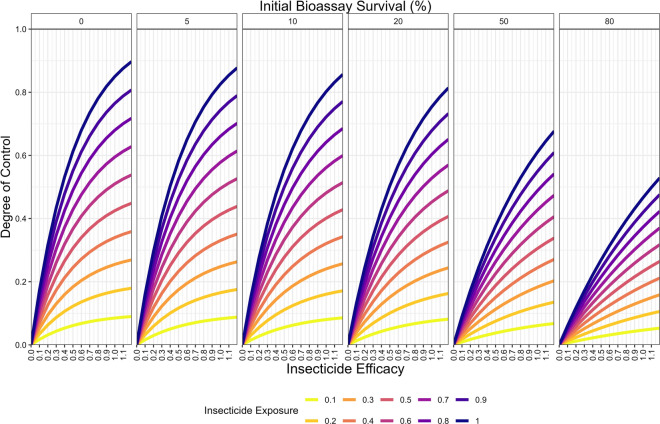
Impact of insecticide efficacy, exposure and resistance on the single generation changes in degree of control for monotherapy deployments. Description is as for Fig. 5 but the y-axis now shows degree of control

Between these two extremes, as the insecticide efficacy decays, there is a period where both a significant proportion of the exposed mosquitoes survive and that these mosquitoes are moderately to very highly resistant (mechanistically shown in Fig. 2: Moderate dose insecticide). The proportion of individuals surviving the insecticide is sufficiently large that it is not largely diluted by the mosquitoes which avoided selection. These increases in the level of insecticide resistance selection are combined with decreases in the ability to control the target mosquito population (Fig. 6). The response to selection is highest under such conditions of intermediate efficacy (Fig. 4), providing some mosquitoes escape selection (if this does not occur, i.e., exposure = 1, then the relationship is strictly increasing; Fig. 5).

### Insecticide decay and mixture deployments: change in insecticide resistance level and control over a single generation

**Fig. 7:**
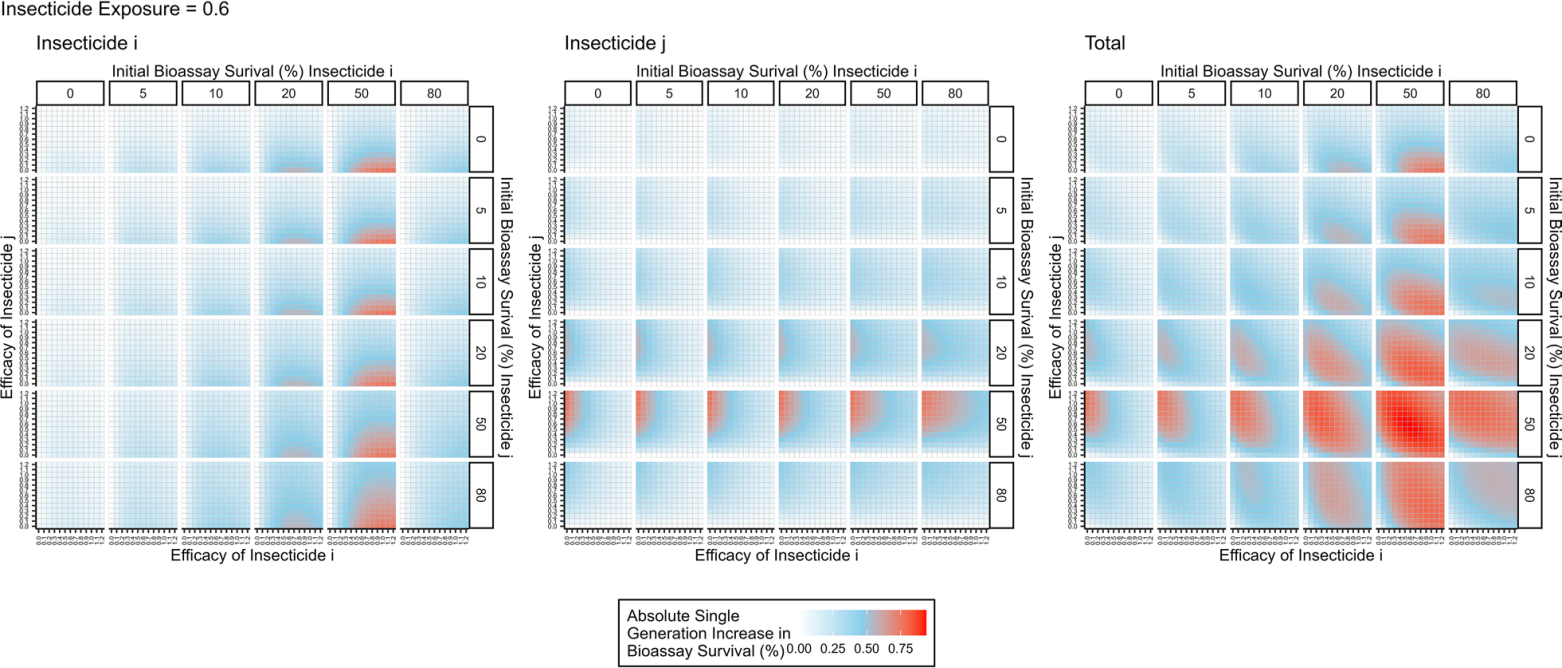
Single generation changes in bioassay survival (%) for each insecticide in the mixture, with exposure = 0.6. Interpretation: Red values indicate larger changes in bioassy survival so are worse for IRM. Pale blue values indicate smaller change in bioassay survival which are better for IRM. Left plot: Change in bioassay survival for insecticide $i$ only. Middle plot: Change in bioassay survival for insecticide $j$ only. Right plot: Total combined change in bioassay survival (change bioassay survival insecticide $i$ + change bioassay survival insecticide $j$). The x axis is the efficacy of insecticide $i$ and y axis is the efficacy of insecticide $j$. Panels (left–right): the amount of initial resistance to insecticide $i$ as measured in a bioassay. Panels (top–bottom): the amount of initial resistance to insecticide $j$ as measured in a bioassay

The next step is to consider the impact of insecticide decay for mixtures. When only considering a single generation there is no need to define a decay profile (e.g., Fig. 3) but assume insecticidal decay has reduced the efficacy of one (or both) insecticides and focus on how reduced efficacy drives IR. Figure 7 is a heat map of the responses to selection for mosquitoes exposed to a mixture of both insecticide $i$ and insecticide $j$ (assuming, for illustration, that 60% of mosquitoes in the population are exposed to the insecticide). Figure 7 contains 3 panels, one for the response to each insecticide separately and a third for the total response (indicating the total amount of selection on the population). This demonstrates how selection acts on resistance to each insecticide.

**Fig. 8:**
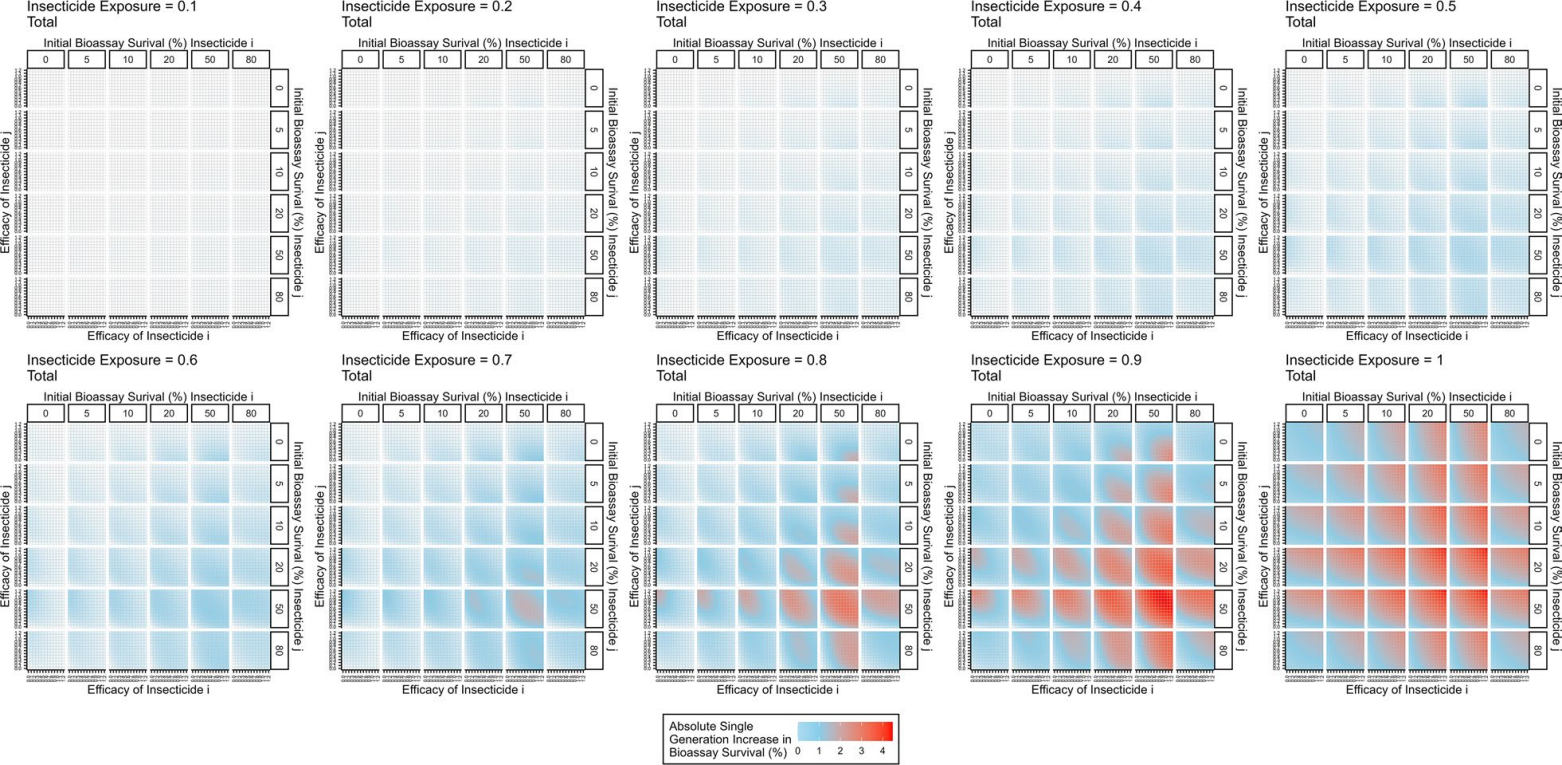
Single generation changes in bioassay survival (%) for each insecticide in the mixture accounting for exposure, resistance and insecticide efficacy. Interpretation: Red values indicate larger changes in bioassy survival so are worse for IRM. Pale blue values indicate smaller change in bioassay survival and so are better for IRM. Total combined change in bioassay survival (change bioassay survival insecticide $\text{i}$ + change bioassay survival insecticide $\text{j}$). The x axis is the efficacy of insecticide $\text{i}$ and y axis is the efficacy of insecticide $\text{j}$. Each major panel is for the level of exposure, within these the mini-panels going left–right is the amount of initial resistance to insecticide $\text{i}$ as measured in a bioassay and mini-panels going top–bottom is the amount of initial resistance to insecticide $\text{j}$ as measured in a bioassay

**Fig. 9:**
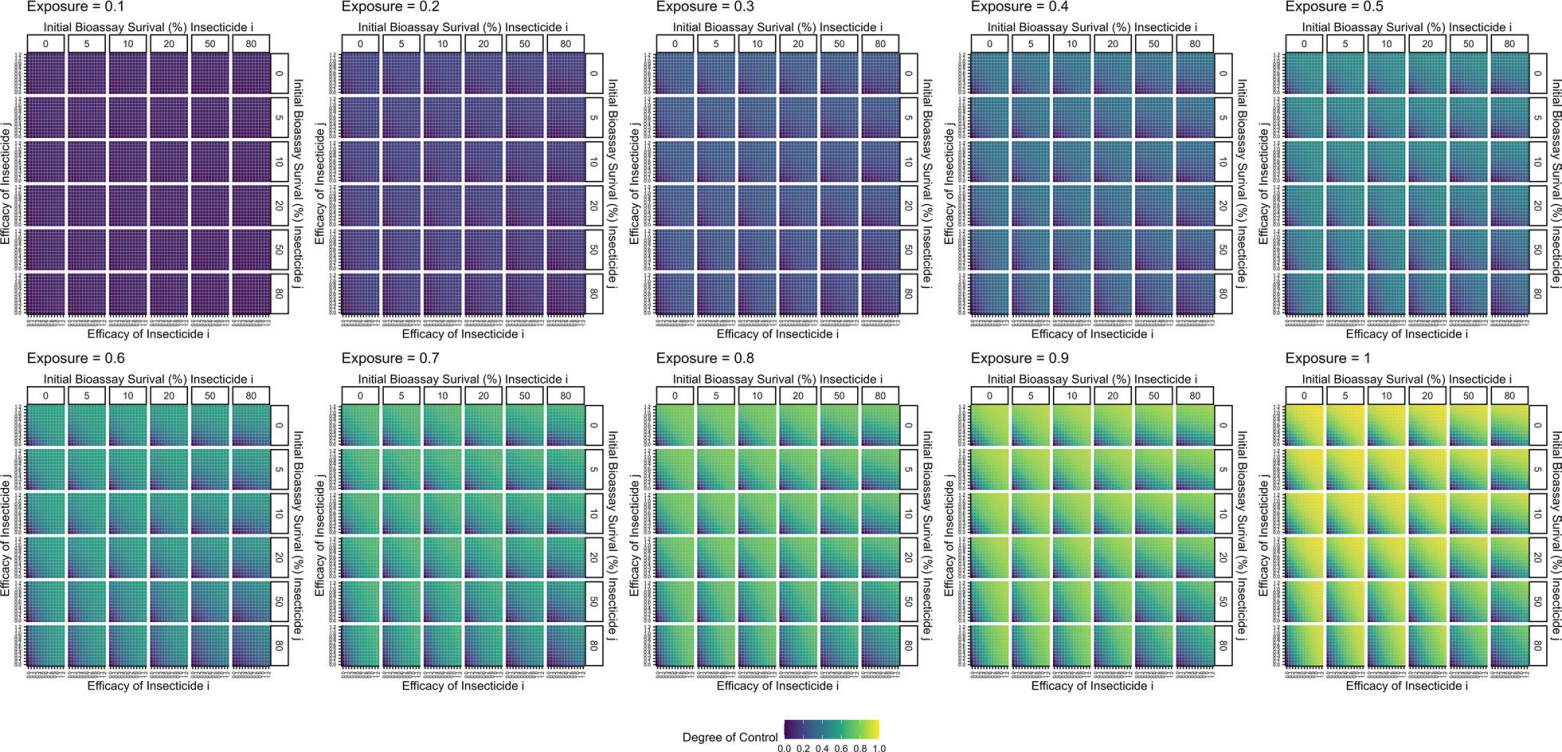
Expected degree of control accounting for exposure, resistance and insecticide efficacy in a single generation. The x axis is the efficacy of insecticide $i$ and y axis is the efficacy of insecticide $j$. Each major panel is for the level of exposure, within these the mini-panels going left–right is the amount of initial resistance to insecticide $i$ as measured in a bioassay and mini-panels going top–bottom is the amount of initial resistance to insecticide $j$ as measured in a bioassay

Figure 8 further explores how changing the exposure impacts insecticide resistance selection. When exposure is 1, no mosquitoes escape exposure and therefore increasing the efficacy always increases the amount of selection, as there is no population of unselected individuals to dilute the populations (as similarly seen for monotherapy deployments). Figure 8 shows that the response to selection is highest where there is already resistance to both insecticides and/or (equivalently) the efficacy of both insecticides is reduced. This can be mechanistically explained using Fig. 4. In this figure, the mortality induced by insecticide $i$ remains constant between each panel, with the additional mortality induced by insecticide $j$ changing. It shows that additional mortality imposed by insecticide $j$ reduces the response to selection for insecticide $i$. This is because insecticide $j$ kills additional mosquitoes in the exposed group and consequently increases the proportion of the final parental population which are from the unexposed group. Of course, there would be simultaneously selection for resistance to insecticide $j$. Figure 5 previously showed that increasing exposure increases the degree of control for monotherapy. A similar result occurred for mixtures i.e., increasing exposure increases the degree of control (Fig. 9).

### Impact of insecticide decay on IRM strategy lifespans: multi-generational changes in insecticide resistance

Simulations were run to investigate the long-term impact of insecticide decay on the performance of mixtures versus monotherapy sequences over operational resistance management timescales (up to 500 mosquito generations, equivalent to 50 years).

**Fig. 10:**
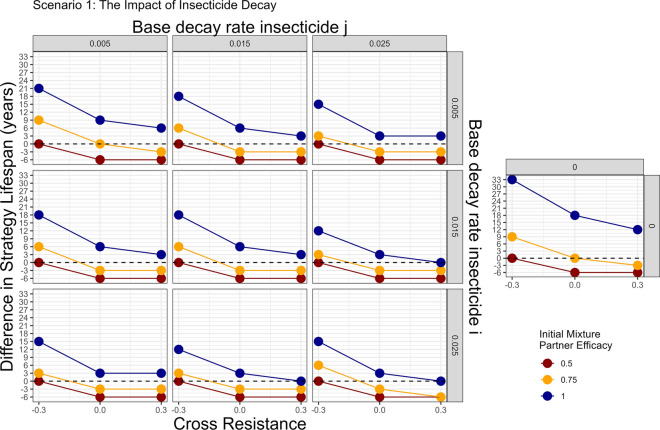
Scenario 1—Impact of including insecticide decay in simulations evaluating mixtures versus monotherapy sequences: two stage decay. The colours indicate the initial efficacy at deployment of each insecticide when in mixture (assumed to be the same for each insecticide). The horizontal dashed line at difference = 0 indicates the mixture strategy and monotherapy sequence strategy had the same strategy lifespans. Values above this line indicate the mixture strategy had a longer strategy lifespan and values below this line indicates the monotherapy sequence strategy had the longer strategy lifespan. The panels top–bottom are the base decay rate for insecticide $i$, and the panels left–right are the base decay rate for insecticide $j$. The x axis (bottom) is the degree of cross resistance between the two insecticides. The inset graph is the simulation where insecticide decay does not occur and has the same axis labels

Scenario 1 (Table 3; Fig. 10) focusses on insecticide decay, without the additional complexities of mis-matched initial resistance or mis-matched heritability. Failure to include insecticide decay appears to overestimate the effectiveness of full-dose mixtures for IRM when compared to monotherapy sequences, albeit these full-dose mixtures still outperform the monotherapy sequence strategy. At higher rates of decay, the benefit of full-dose mixtures became smaller. The reduced dose mixtures performed poorly regardless of whether insecticide decay was included or not.

**Fig. 11:**
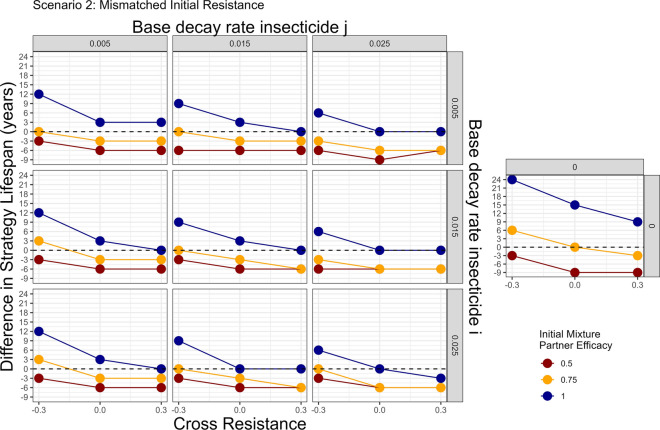
Scenario 2—Impact of including insecticide decay in simulations evaluating mixtures versus monotherapy sequences with mismatched initial resistances: two stage decay. The colours indicate the deployed efficacy of each insecticide when in mixture. The horizontal dashed line indicates a difference in lifespan of zero i.e. the mixture strategy and monotherapy sequence strategy had the same strategy lifespans. Values above this line indicate the mixture strategy had a longer strategy lifespan and values below this line indicates the monotherapy sequence strategy had the longer strategy lifespan. The panels top–bottom are the base decay rate for insecticide $i$, and the panels left–right are the base decay rate for insecticide $j$. The x axis (bottom) is the degree of cross resistance between the two insecticides. The inset graph is the simulation where insecticide decay does not occur and has the same axis labels

**Fig. 12:**
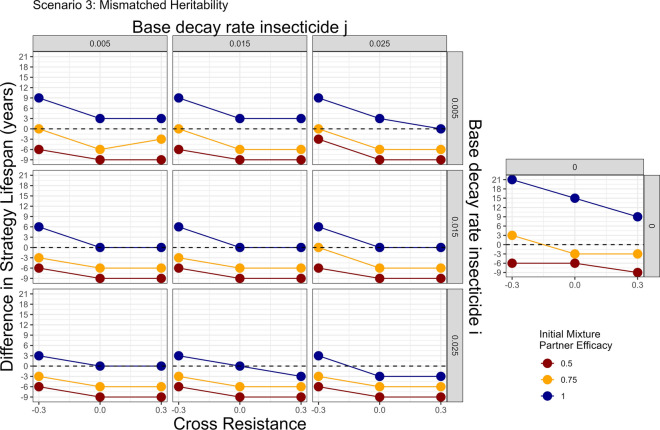
Scenario 3—Impact of including insecticide decay in simulations evaluating mixtures versus monotherapy sequences with mismatched heritabilities: two stage decay. In this scenario the two insecticides had different heritabilities. The heritability for insecticide $i$ (${h}_{I}^{2}$) was 0.15 and the heritability for insecticide $j$ (${h}_{J}^{2}$) was: 0.25. The colours indicate the deployed efficacy of each insecticide when in mixture. The dashed line indicates the mixture strategy and monotherapy sequence strategy had the same strategy lifespans. Values above this line indicate the mixture strategy had a longer strategy lifespan and values below this line indicates the monotherapy sequence strategy had the longer strategy lifespan. The panels top–bottom are the base decay rate for insecticide $i$, and the panels left–right are the base decay rate for insecticide $j$. The x axis (bottom) is the degree of cross resistance between the two insecticides. The inset graph is the simulation where insecticide decay does not occur and has the same axis labels

Scenario 2 (Table 3; Fig. 11) extends Scenario 1 to allow two insecticides with mis-matched initial resistance. Concerningly, when there was some pre-existing resistance to one of the mixture insecticides there are decay profiles where the full-dose mixture performs worse than monotherapy sequences. Scenario 3 (Table 3; Fig. 12) extends Scenario 1 by allowing mis-matched heritabilities for the resistance traits. Again, there are decay profiles where full-dose mixtures perform worse than monotherapy sequences.

The generalities across these three scenarios are that (a) failure to include insecticide decay consistently overestimates the benefit of full-dose mixtures (b) reducing the dose each partner in a mixture is additionally concerning because this can make the mixtures less viable than monotherapy sequences (c) combining these two factors is especially concerning. These generalities remain when relaxing the assumption of a two-stage decay rate and instead assume insecticide efficacy decays at a constant rate (Supplement 3).

## Discussion

Insecticide decay is frequently highlighted as an important consideration in the deployment of insecticides in public health, especially with regards to mixtures [18]. Despite this putative importance, insecticide decay has been missing from most models [12], a limitation often noted when investigating mixtures [19, 20]. A dynamic model of polygenic insecticide selection [31] was used here to investigate the likely consequences of this omission, under both single- and multi-generation selection scenarios considering both monotherapy and mixture deployments.

### Impact of insecticide decay on monotherapies

Monotherapy deployments (such as using pyrethroid only ITNs) can include periods post-deployment where the overall selection for resistance is higher than when the insecticide was newly deployed (Fig. 5). Obviously, the key consideration is the duration of these periods which depends on the decay profile of the specific insecticides (Fig. 3). A similar result was found when modelling monogenic systems [12].

### Impact of insecticide decay on mixtures

The impact of insecticide decay on the performance of mixtures as an IRM strategy has long been highlighted as an important knowledge gap [18]. Figures 7 and 8 show how the rate of selection for resistance to one insecticide in a mixture is modulated by the efficacy and resistance status of the partner insecticide. Perhaps the most concerning result is that the rate of the selection (to the mixture as a whole) appears to be fastest when both insecticides are at lower efficacies; worryingly, this may be the approximate efficacy of half-dose mixtures. A similar result was obtained from simulations assuming a single-gene basis of resistance [23] where it was concluded that this likely arose because strong levels of mutual protection in mixtures are required to maximize their lifespan (i.e. if an insect is resistant to one insecticide in the mixture it must be reliable killed by the second). This is a plausible explanation for the results presented here i.e., high levels of pre-existing resistance to one insecticide, or decaying effectiveness post-deployment reduces this level of mutual protection in mixtures. This substantially reduces their superiority over sequential monotherapy deployments. The decay profile of mixtures in the field will of course determine how long they spend at concentrations where selection is higher or lower; and worryingly these reductions in insecticide efficacy are likely to coincide with reductions in disease control. Operationally, the requirement is the timely replacement of deployments that have become suboptimal by programmes such as periodic reapplication of IRS or replacement of LLINs. An interim IRM strategy may be to increase the rate at which worn-out standard pyrethroid-only LLINs are replaced with new ones. Damaged nets can be checked through visual observation [37], to identify LLINs where there is no longer sufficient insecticide is more challenging.

### Impact of insecticide decay on IRM strategy performance

Insecticide decay occurs regardless of the IRM strategy used. However, the important question is whether the inclusion of insecticide decay in simulations change the choice of which IRM strategy should be deployed? The results show that this is an important issue for impacting the rate of selection (Figs. 5 and 8) and is also important in determining whether one strategy is more effective than another (Figs. 9, 10 and 11). The failure to include insecticide decay in previous models evaluating mixtures versus monotherapies (e.g., [13, 21, 23, 25, 28]) may unfortunately have been giving overly optimistic estimates of the performance of full-dose mixtures for IRM. Levick et al. [23] did include pre-existing resistance in their simulations and noted that mixtures became less effective or even worse that sequential monotherapy (e.g. their Fig. 8); by inference, it is likely that the same effect would occur if pre-existing resistance was replaced by decayed effectiveness reducing effectiveness. This was recognized and noted by these authors but the work presented here is the first to explicitly include the effect of insecticide decay on the entire post-deployment time-period of mixtures and monotherapies.

### Caveat to IRM models: degree of control as an evaluation measure

These mechanistic descriptions of how mixtures (Fig. 4) work also help demonstrate the additional kill concept [26], e.g., the orange parts of Fig. 4. Mixtures (especially when both insecticides are at high dose) would be expected to kill more individuals than monotherapies and this benefit of increased population suppression is not usually considered in the evaluation of IRM strategies [26]. This is, especially a problem when evaluating public health interventions as converting resistance into an impact on disease transmission is a complex challenge [38]. Additionally Fig. 4 shows how mixtures cause an additional decrease in the population size of mosquitoes post treatment (likely providing an increased vector control benefit). Survivors of the mixture then constitute a small proportion of the final adult population. That is, adults which survive the mixture are few, and are diluted by those unexposed to the insecticide (and were not exposed to selection), and this dilution by unselected individuals provides the IRM benefit.

The inclusion of an intervention efficacy-decay parameter in disease transmission models is common e.g., [39–41] and used to account for the expected reduction in personal protection and reduced impact on disease transmission over time. Often these parameters account for insecticide decay, reduced coverage and the increase in the holes in nets (which reduce their efficacy) over time. However, these decaying insecticides are likely still selecting for insecticide resistance further compromising long-term personal protection and disease transmission. Agent-based transmission models used for evaluating complex malaria control interventions do not yet include insecticide resistance evolution (they usually include resistance as a fixed effect (e.g. [42])), with the notable exception of EMOD [43].

### A composite measure of IRM and vector control

The deployment of insecticides for use in public health is primarily to kill mosquitoes (to reduce disease transmission). However the widespread evolution of resistance has forced a secondary aim i.e., that they are deployed in such a way which reduces the selection for resistant mosquitoes to maintain the longer-term sustainability of the intervention. Madgwick and Kantiz [27] highlighted the concept of “control time” which accounts for both the “degree of control” and “degree of IRM”, although with a greater focus on agriculture. Figures 6 and 9 show that deploying insecticides such that when exposure is higher (which could be achieved through increased coverage or better targeting of local mosquito behaviours) results in a higher level of expected “degree of control”, although this unfortunately generally comes at the expense of increased levels of selection on resistance (Figs. 5 and 8). The key operational question is therefore how to maximize the “degree of control” so as to reduce disease transmission while simultaneously minimizing the response to selection. This would potentially ensure that effective control is viable until disease elimination has been achieved. For mosquitoes (and, more specifically, those species responsible for malaria transmission), the dynamics of mosquito population size regulation need to also consider the age-structuring [44] as female mosquitoes must survive the extrinsic incubation period to become infectious (malaria generally takes > 10 days to develop in mosquitoes so only older mosquitos can transmit the disease).

## Conclusion

Insecticide effectiveness at deployment *and* their subsequent decay in efficacy are key considerations when evaluating IRM strategies. The latter has often been neglected despite being frequently highlighted as an issue. As shown above, as insecticides decay, there can be substantial changes in the rate of selection for resistance. The absence of insecticide decay from previous models appears to have been providing overly optimistic estimates of the performance of full-dose mixtures versus monotherapy deployments, although full-dose mixtures generally still outperform sequential monotherapies. The main impact of insecticide decay is on mixtures whose constituent insecticides are not deployed at their full doses: decaying effectiveness from a reduced baseline appears to rapidly erode any advantage that mixtures may have over monotherapy deployment (e.g., Fig. 10).

## Supplementary Information

Additional file 1.

Additional file 2.

Additional file 3.

## Data Availability

Model code for the running and analysis of simulations is available from the GitHub repository: https://github.com/NeilHobbs/polytruncate and the corresponding author upon request.

## Abbreviations

LLIN: Long-lasting insecticidal nets
IRM: Insecticide resistance management
IRS: Indoor residual spraying
ITN: Insecticide-treated net
PRS: Polygenic resistance score
WHO: World Health Organization
