# Supplementary material for: The impact of insecticide decay on the rate of insecticide resistance evolution for monotherapies and mixtures
Source: Malar J. 2025 Feb 18;24:50. doi: 10.1186/s12936-024-05147-y (PMC11837469; doi:10.1186/s12936-024-05147-y)
Supplement: Supplementary file 1 — Additional file 1. [file 12936_2024_5147_MOESM1_ESM.docx]

**Supplement 1: Estimation of Insecticide Decay Rates for LLINs:**

For the insecticide decay profiles we provide default values for an LLIN insecticide. We note that insecticide decay is likely to be highly heterogenous between different locations due to climatic conditions and that local-level conditions will also play a role. This is perhaps most notable for IRS where the substrate the insecticide is sprayed on has a large effect on how fast or slow the efficacy of the insecticide declines. Our approach is therefore to construct realistic default, illustrative decay profiles, rather than attempting to fit decay profiles to specific insecticides under specific conditions. We use the parameter values estimated as a default baseline and investigate the impact of the insecticide decaying faster/slower and/or rapidly decaying sooner/late. This enables more general global conclusions to be assessed. We use data reported by Toé et al (2019), on field collected LLIN assessed in wire ball assays against a fully susceptible pyrethroid susceptible strain (Kisumu; Bioassay Survival = 0%). LLIN collections from the field were made at 0, 6, 12, 18 and 24 months post deployment (Toé et al., 2019). In our mosquito generation scale this correspond to 0, 5, 10, 15 and 20 mosquito generations respectively (we assume 10 mosquito generations per year). As initial wire ball bioassay survival was ~10% we converted the initial starting insecticide efficacy to 0.9. Equation 1b(ii) from Hobbs & Hastings (2024) was used to estimate the base decay rate, rapid decay rate and threshold generation. A broadly matching decay profile was achieved with base decay = 0.015, rapid decay = 0.08 and threshold generations = 15 (Figure S1.1). These values are therefore the default decay rates and decay thresholds. We can therefore vary these values to assess how the speed (and shape) of the decay profile impacts the rate of insecticide resistance evolution, and what the implications are of insecticides which decay faster and/or slower.


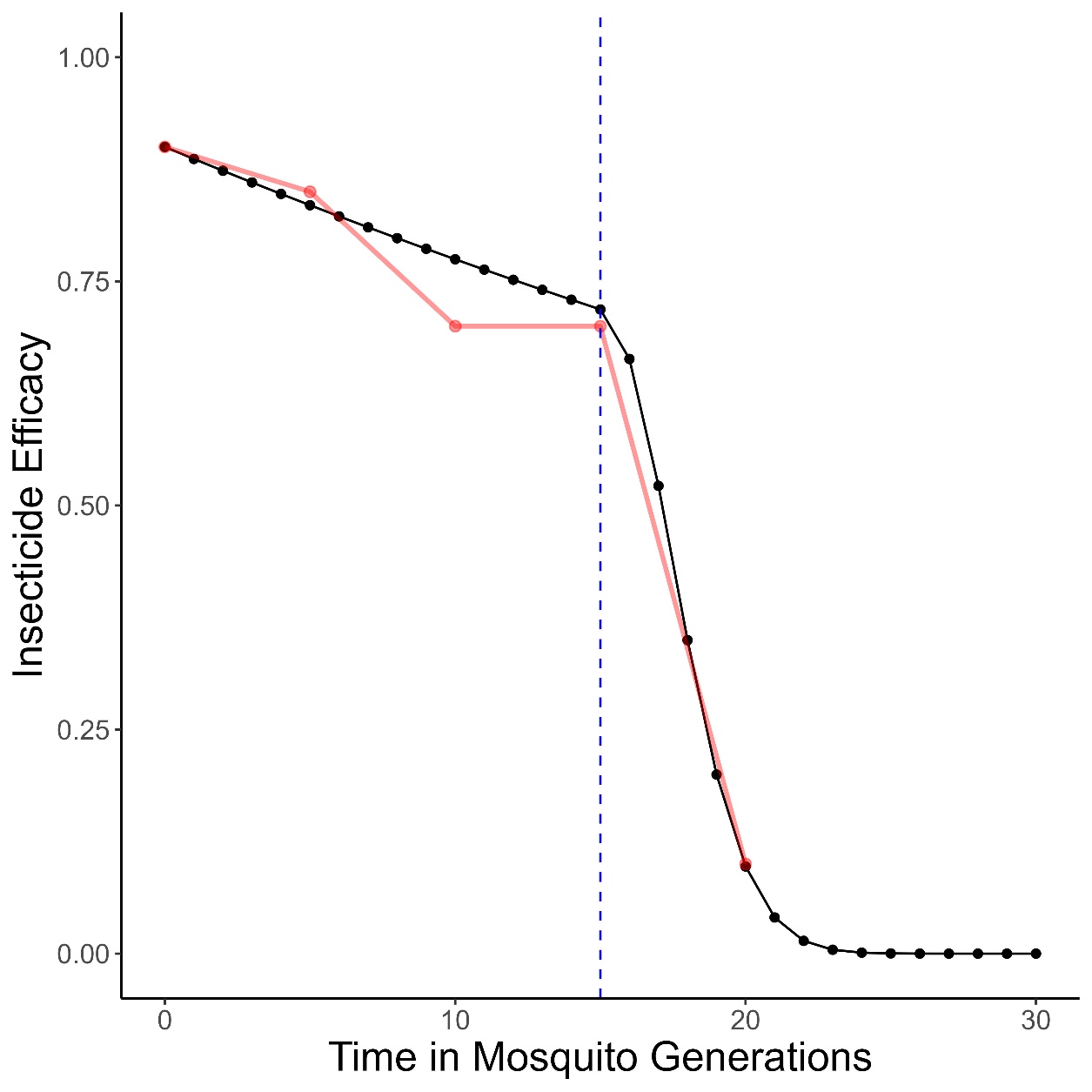
**Figure S1.1: Estimating Insecticide Decay Rates.** Red dots and line is the measured insecticide efficacy from wire-ball assays from field collected LLINs over time. Black line is calculated insecticide efficacy with a base decay rate of 0.015, a rapid decay rate of 0.08 and a threshold generation of 15 (~1.5 years).

**References:**

Hobbs, N. P., & Hastings, I. M. (2024). Mathematical Methodology for Dynamic Models of Insecticide Selection Assuming a Polygenic Basis of Resistance. *BioRxiv*, *[Preprint]*, 1–67. https://doi.org/https://doi.org/10.1101/2024.04.30.591816

Toé, K. H., Mechan, F., Tangena, J. A. A., Morris, M., Solino, J., Tchicaya, E. F. S., Traoré, A., Ismail, H., Maas, J., Lissenden, N., Pinder, M., Lindsay, S. W., Tiono, A. B., Ranson, H., & Sagnon, N. (2019). Assessing the impact of the addition of pyriproxyfen on the durability of permethrin-treated bed nets in Burkina Faso: A compound-randomized controlled trial. *Malaria Journal*, *18*(1), 1–16. https://doi.org/10.1186/s12936-019-3018-1
