# Supplementary material for: The impact of insecticide decay on the rate of insecticide resistance evolution for monotherapies and mixtures
Source: Malar J. 2025 Feb 18;24:50. doi: 10.1186/s12936-024-05147-y (PMC11837469; doi:10.1186/s12936-024-05147-y)
Supplement: Supplementary file 2 — Additional file 2. [file 12936_2024_5147_MOESM2_ESM.docx]

**Supplement 2: An Overview to the Polytruncate Model**

The model presented in [31], is a complex and dynamic model. We therefore present in an accessible manner the mathematical methodology of how the truncation selection process in the model works. We use Figure S2 as a conceptual figure to help illustrate the insecticide selection process and relate these to the equations. Readers requiring further technical details are directed to [31] as mentioned in the main manuscript. We describe the model across four key points.


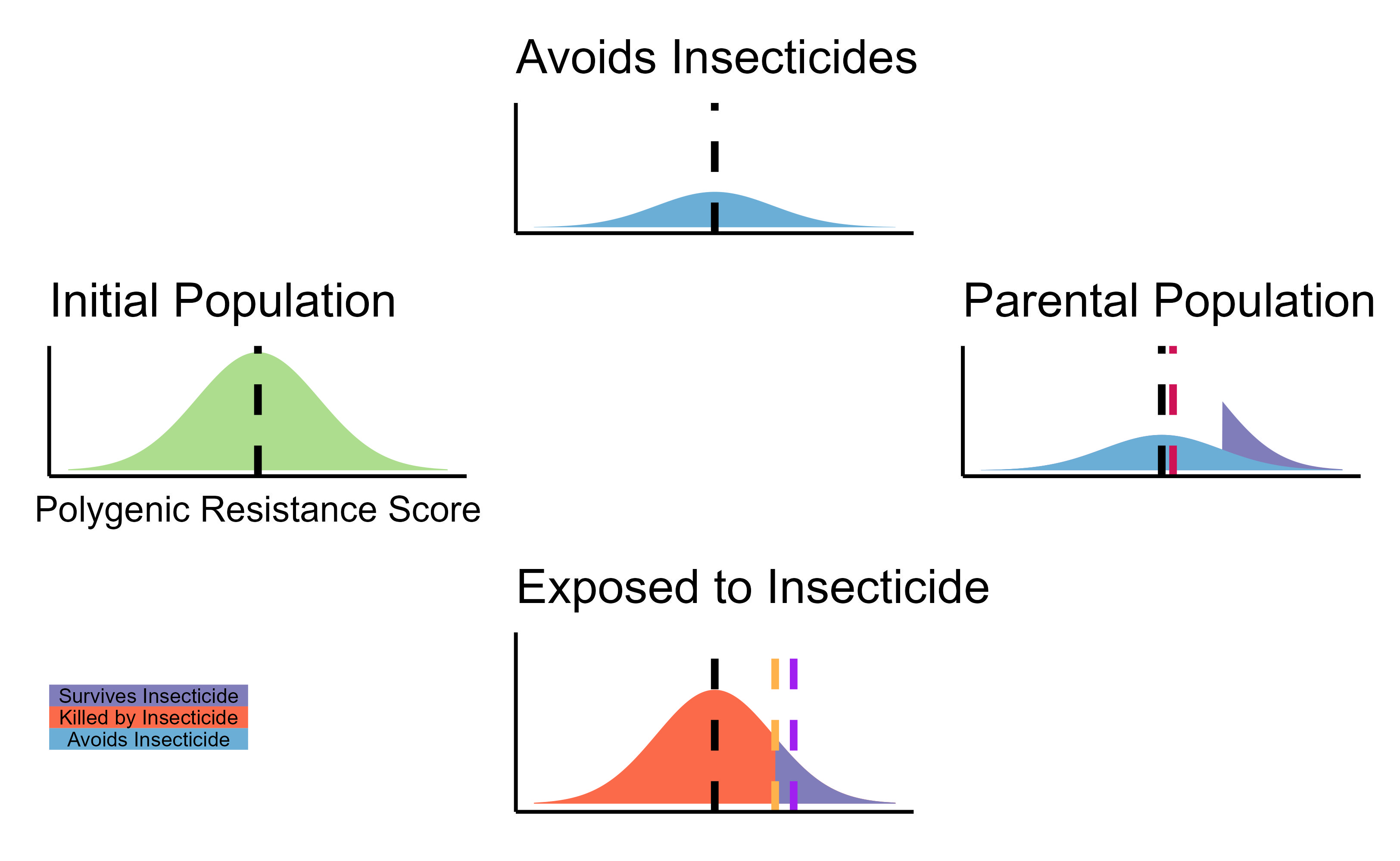


**Figure S2: Single Generation Insecticide Resistance Selection Process with Truncation Selection.** Mosquitoes emerge as the initial population (for the generation) and have a mean polygenic resistance score (PRS) (indicated by the black dashed line), as shown in the left panel, “Initial Population”. These mosquitoes are either exposed to the insecticide (bottom panel, “Exposed to Insecticide”), or are not exposed to the insecticide (top panel, “Avoids Insecticide”). The “Exposed to Insecticide” group undergo insecticide induced mortality, where the amount of mortality will depend on both the level of resistance in population and the efficacy of the population, only mosquitoes whose PRS is higher than the required threshold selection value (dashed orange line) will survive the exposure, this group of mosquitoes then have an updated mean PRS (dashed purple line). The final parental population (right panel) is the both those who survived the insecticide and those who avoided it. The final parental population has an updated mean PRS (red dashed line). The selection differential is then the red dashed line minus the black dashed line. Note, this process is conducted separately for female and male mosquitoes, such that there are sex-specific selection differentials.

**Point 1: Defining the Polygenic Resistance Score**

The model tracks a mean polygenic resistance score over successive generation, which describes the “amount of resistance” in the mosquito population. We convert the polygenic resistance score to bioassay survival using the Hill-variant of the Michaelis-Menten equation, which converts the PRS to survival in a WHO cylinder bioassay:

$$K_{i}^{B}=\frac{K_{max}*{z_{I}}^{n}}{z_{50}+{z_{I}}^{n}}$$

Equation S1

This therefore allows us to report the polygenic resistance score in an interpretable format. We scale this equation such that a polygenic resistance score of 100 corresponds to a 10% bioassay survival score, and therefore the $z_{50}$ is 900.

**Point 2: Selection Differentials and Response to Selection**

The model uses a quantitative genetics approach to model resistance as a polygenic trait. Therefore the sex-specific Breeder’s equation is:

$$R_{I}^{S\phi}=h_{I}^{2} \frac{(S_{I}^{S\phi\text{♀}}+S_{I}^{S\phi\text{♂}})}{2}\beta$$

 Equation S2

This equation is identical to that in the manuscript (Equation 1), except replacing the words with the corresponding symbols. $R_{I}^{S\phi}$ (the Response to Selection) is the between generation change in the polygenic resistance score. $S_{I}^{S\phi\text{♀}}$ and $S_{I}^{S\phi\text{♂}}$ are the selection differentials and correspond to the change in the mean polygenic resistance pre-selection (black dashed line, Figure S2) and post selection (red dashed line, Figure S2). Our methodology [31] proposes that the selection differentials are calculated based on the level of resistance in the population (i.e., the polygenic resistance score) and the efficacy of the insecticide. That is how many mosquitoes will survive the insecticide exposure will depend on both these factors,

**Point 3: Describing Insecticide Efficacy**

The ability of an insecticide to kill a mosquito will depend on the dose of that insecticide, the duration the mosquito contacts the insecticide for, and the bioavailability of the insecticide. Bioavailability will be different between ITN brands or IRS formulations for example. For ITNs, the net contact duration is likely to decrease as the physical net structure itself degrades. To account for this issue between different manufactures and dosing, we define the manufacturers specified dose having an insecticide efficacy ($\omega_{0}^{i}$) of 1, against a fully susceptible mosquito population. This is analogous to the LD100 value for the insecticide against a fully susceptible mosquito population.

We make the assumption that insecticide decay (especially ITNs) may be a two-stage process. Firstly, insecticides decay rather slowly

$$\omega_{\tau}^{i}= \omega_{0}^{i}*\exp\left( -\tau^{2}* {\delta_{b}}^{i} \right), where 0\leq\tau\leq\tau_{b}^{i}$$

Equation S3

Followed by a period where the insecticide may decay more rapidly, due for instance the physical net structure becoming sufficiently holed that that mosquitoes are able to fly through with potentially only very brief net contact durations.

${\omega_{\tau}^{i}= (\omega}_{0}^{i}* exp(-{\tau_{b}^{i}}^{2}* {\delta_{b}}^{i}))* \exp\left( -(\tau- \tau_{b}^{i} \right)^{2}* {\delta_{r}}^{i}), \mathrm{where}\tau_{b}^{i}< \tau$ Equation S4

The proportion of mosquitoes surviving the insecticide exposure is therefore:

$$K_{i}^{F}= {{(\varphi}_{1}K_{i}^{B}+\varphi_{2})}^{\omega_{\tau}^{i}}$$

Equation S5

$K_{i}^{F}$ is then the proportion of mosquitoes which survive the insecticide (i.e., the area of the purple shaded region). Readers will note that when $\omega_{\tau}^{i}$=1, this is identical to the format used in [21], as this was the assumption in that model. The values ${\delta_{b}}^{i}$(base decay rate) and ${\delta_{r}}^{i}$ (rapid decay rate) are in insecticide per generation.

**Point 4: Calculating the Selection Differentials Mechanistically**

Here we describe the truncation selection process for female mosquitoes. An analogous process also occurs for male mosquitoes.

$$S_{I}^{S\text{♀}}= \bar{z}_{I}^{P\text{♀}}- \bar{z}_{I}^{\text{♀}}$$

Equation S6

The value $S_{I}^{S\text{♀}}$ is the insecticide selection differential and is the value calculated each generation, dependent on the level of resistance in the population (i.e., the polygenic resistance score) and the efficacy of the insecticide. $\bar{z}_{I}^{\text{♀}}$ is the Mean PRS of the mosquito population before selection.

$\bar{z}_{I}^{P\text{♀}}$ is the mean polygenic resistance score of the parents (red dashed line, Figure S2), and to calculate this we need to know: First the number surviving the insecticide (purple shaded area, Figure S2) and their mean polygenic resistance score (purple dashed line, Figure S2). And second how many mosquitoes escaped selection (blue shaded area, Figure S2) and their mean polygenic resistance score (black dashed line, Figure S2, remains unchanged as no insecticide selection).

$$\bar{z}_{I}^{P\text{♀}}= \frac{{N_{i}^{E\text{♀}}\bar{z}}_{I}^{E\text{♀}}+ N^{u\text{♀}}\bar{z}_{I}^{\text{♀}}}{N^{P\text{♀}}}$$

 Equation S7

Relating these symbols to their representation on Figure S2: $N_{i}^{E\text{♀}}$ is the purple shaded area, $\bar{z}_{I}^{E\text{♀}}$ is the purple dashed line, $N^{u\text{♀}}$ is the light blue shaded area and $N^{P\text{♀}}$ is the purple shaded area and the light blue shaded area.

The total number of mosquitoes in the parental population is then the sum of the two: $N^{P\text{♀}}= N_{i}^{E\text{♀}}+ N^{u\text{♀}}$ Equation S8

The number of mosquitoes not exposed to the insecticide is the proportion not exposed:

$$N^{u\text{♀}} = N^{T\text{♀}}\left( 1-x \right)$$

 Equation S9

The mean polygenic resistance score of the exposed survivors is the mean polygenic resistance score of the population before selection plus the selection differential between them and the survivors.

$$\bar{z}_{I}^{E\text{♀}}= \bar{z}_{I}^{\text{♀}}+S_{I}^{E\text{♀}}$$

Equation S10

Unfortunately $\bar{z}_{I}^{E\text{♀}}$ is unknown, but $S_{I}^{E\text{♀}}$ can be calculated using standard truncation selection approaches:

$$S_{I}^{E}= \sigma_{I}\frac{\varphi\left( {\bar{z}_{I}}_{[1-\bar{K}_{i}^{F}]} \right)}{\bar{K}_{i}^{F}}$$

Equation S11

The value $\sigma_{I}$ is the standard deviation. $\varphi(z_{I})$ is the unit Normal density distribution function:

$$\varphi\left( z_{I} \right)= \frac{1}{\sqrt{2\pi}}{exp}^{-z_{I}^{2}/2}$$

Equation S12

Finally the number of mosquitoes that are exposed and survived is:

$$N_{i}^{E\text{♀}}=xN^{T\text{♀}}\bar{K}_{i}^{F}$$

 Equation S13

And for mixtures, where the mosquitoes would have to survive contact with both insecticides.

$$N_{i}^{E\text{♀}}=xN^{T\text{♀}}\bar{K}_{i}^{F}\bar{K}_{j}^{F}$$

Equation S14

| **Table S2.1 Parameters and Descriptions** | | |
| --- | --- | --- |
| Parameter Symbol and Name | Description | Corresponding location on Figure S2. |
| $\bar{z}_{I}^{\text{♀}}$ | Mean poygenic resistance score of the mosquito population. | Dashed black line |
| $K_{i}^{F}$ | Survival probability in the field given insecticide efficacy and the polygenic resistance score. | Not shown on Figure S2. |
| $\bar{z}_{I}^{P\text{♀}}$ | The mean polygenic resistance score of the mosquitoes which will go on to become parents of the next generation. | Dashed red line. |
| $S_{I}^{S\text{♀}}$ | The selection differential as a result of the insecticide. | The difference between the red dashed line and the black dashed line. |
| $\tau$ | Time (in mosquito generations) since the insecticide was last deployed. | Not shown on Figure S2. |
| $\tau_{b}^{i}$ | The number of mosquito generations the insecticide has the base decay rate. | Not shown on Figure S2. |
| ${\delta_{r}}^{i}$ | The rapid decay rate of the insecticide | Not shown on Figure S2. |
| ${\delta_{b}}^{i}$ | The base decay rate of the insecticide. | Not shown on Figure S2. |
| $\omega_{0}^{i}$ | The deployed efficacy of the insecticide. | Not shown on Figure S2. |
| $\omega_{\tau}^{i}$ | The efficacy of the insecticide $\tau$ generations after deployment. | Not shown on Figure S2. However, its impact would be on where the truncation selection occurs (orange dashed line), where higher efficacies shifts to the right, and lower efficacies shift to the left. |
| $N_{i}^{E\text{♀}}$ | The number of mosquitoes which survived the insecticide exposure. | The purple shaded area. |
| $\bar{z}_{I}^{E\text{♀}}$ | The mean polygenic resistance score of the mosquitoes which survived the insecticide exposure. | Purple dashed line. |
| $N^{u\text{♀}}$ | The number of mosquitoes which were not exposed to the insecticide (i.e., escaped selection). | Light blue area |
| $N^{P\text{♀}}$ | The total number of adults in the parental population. | Total area in the final plot |
| $x$ | The probability of encountering the insecticide. | Describes what proportion of the mosquitoes will encounter the insecticide. |
| $N^{T\text{♀}}$ | The total number of mosquitoes before insecticide selection. | Green shaded area |
| $S_{I}^{E\text{♀}}$ | The insecticide selection differential between the exposed survivors and the initial population. | Difference between the purple dashed line and the black dashed line. |
| ${\bar{z}_{I}}_{[1-\bar{K}_{i}^{F}]}$ | The PRS value which is the threshold selection value, above which mosquitoes survive the insecticide. | Orange dashed line |
| $\sigma_{I}$ | The standard deviation of the mean polygenic resistance score. | Describes the shape of the Normal distribution. |
