# Supplementary material for: The impact of insecticide decay on the rate of insecticide resistance evolution for monotherapies and mixtures
Source: Malar J. 2025 Feb 18;24:50. doi: 10.1186/s12936-024-05147-y (PMC11837469; doi:10.1186/s12936-024-05147-y)
Supplement: Supplementary file 3 — Additional file 3. [file 12936_2024_5147_MOESM3_ESM.docx]

**Supplement 3: Changing the decay assumption to a constant linear decay**

Here we perform Scenarios 1, 2 and 3 as previously detailed, however this time with the assumption that insecticide decay is a constant linear process (Figure S3.1) and not the two-stage process as previously examined. The input parameters are the same, except the decay rates are single-stage i.e. the insecticides decay at the base decay rate for the duration of their deployment. This is achieved by setting the threshold decay degeneration to be the same as the deployment frequency.


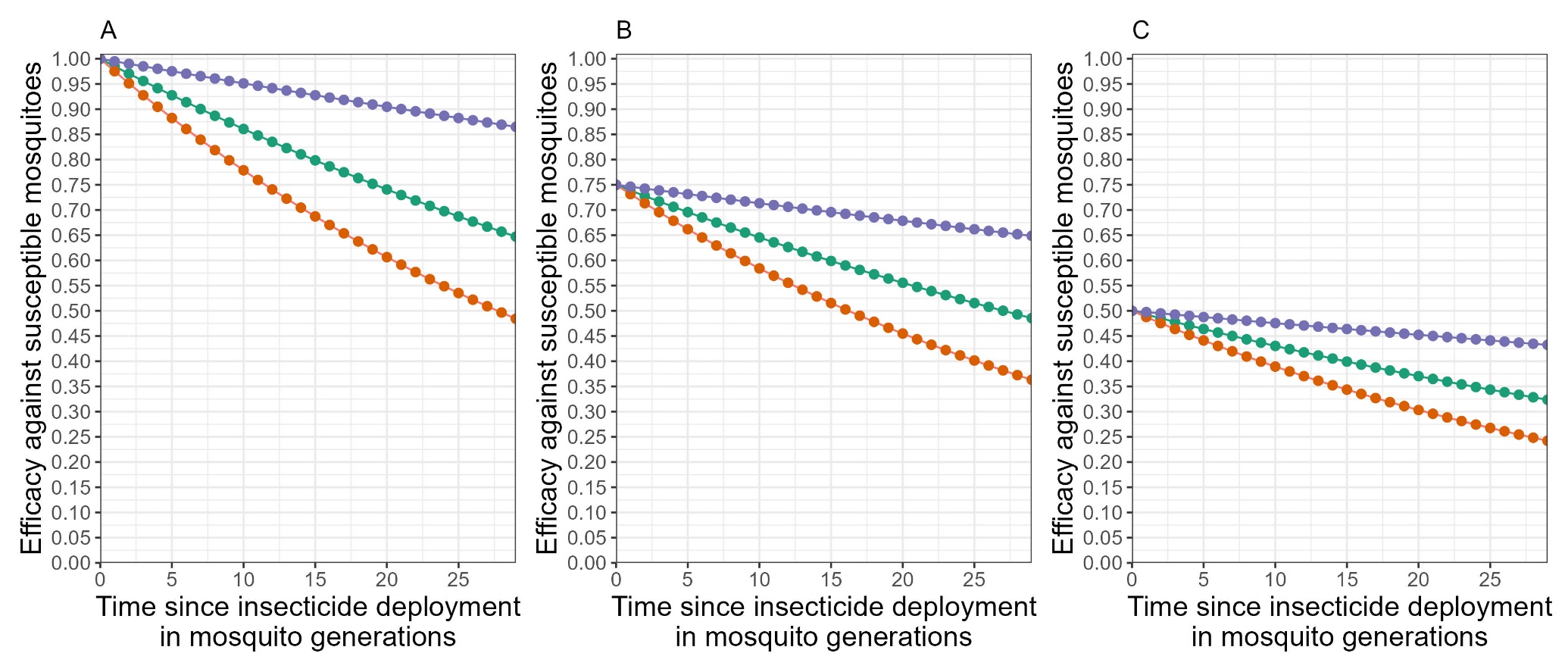


**Figure S3.1** **Example Insecticide Decay Profiles for LLINs assuming a Constant Decay.**  After insecticides are deployed, their efficacy will decrease over time (i.e., “decay,”). The colour of the lines indicates the base decay rate of the insecticide which occurs throughout the deployment and is 0.005 for purple, 0.015 for green (estimated default) and 0.025 for orange, this is the period during which there is slower decay. There is no rapid decay. Panel A is when the insecticide is deployed at the recommended dose, as would occur with monotherapy deployments and full-dose mixtures. Panel B and C are when the insecticide is deployed at a reduced dose as may occur with mixture deployments, and the reduced dose results in the initial efficacy being reduced to either 0.75 (Panel B) or 0.5 (Panel C).


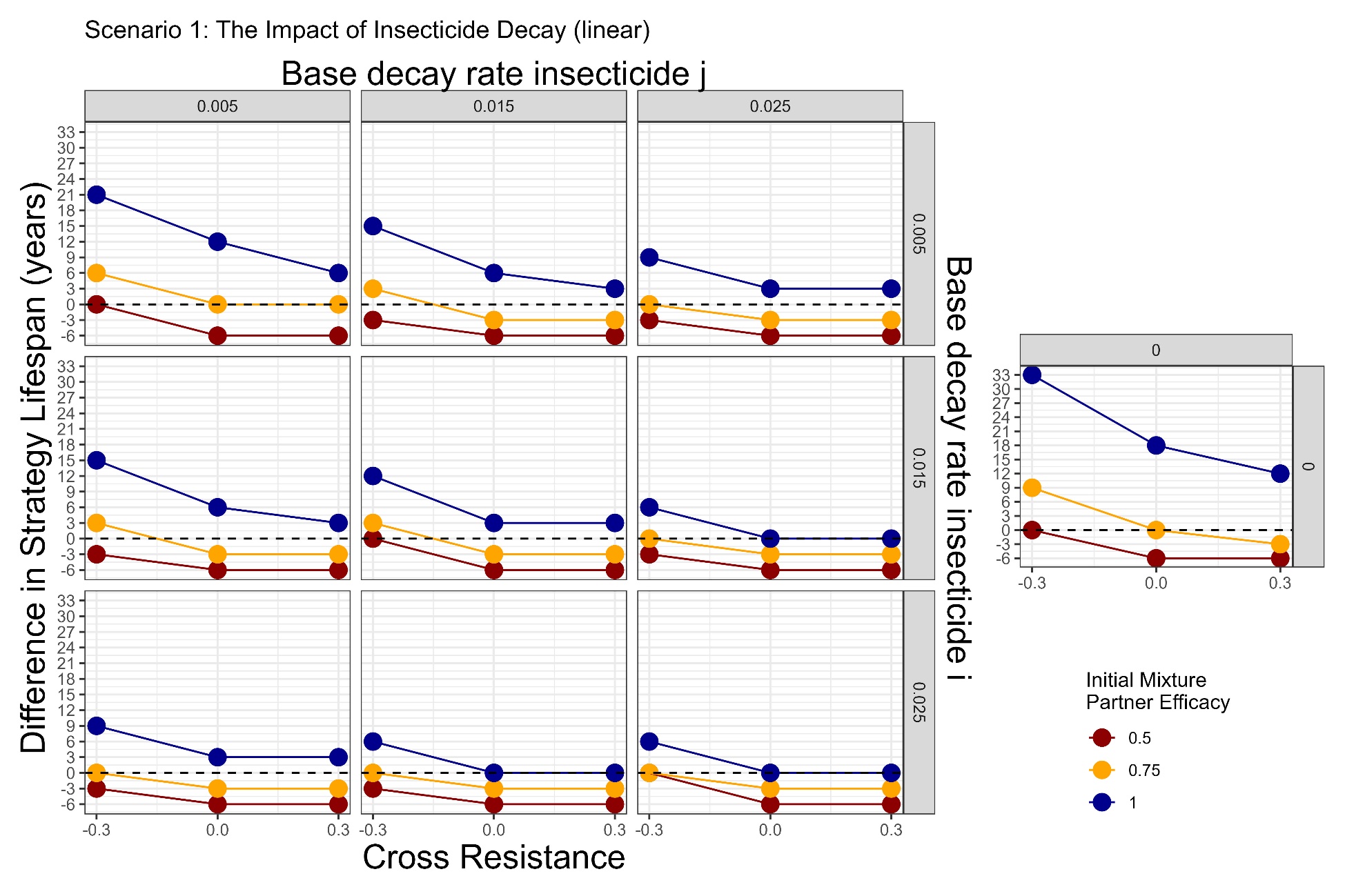


**Figure S3.2 Scenario 1 – Impact of including insecticide decay in simulations evaluating mixtures versus monotherapy sequences: Constant decay.** The colours indicate the initial efficacy at deployment of each insecticide when in mixture (assumed to be the same for each insecticide). The horizontal dashed line at difference=0 indicates the mixture strategy and monotherapy sequence strategy had the same strategy lifespans. Values above this line indicate the mixture strategy had a longer strategy lifespan and values below this line indicates the monotherapy sequence strategy had the longer strategy lifespan. The panels top-bottom are the base decay rate for insecticide $i$, and the panels left-right are the base decay rate for insecticide $j$. The x axis (bottom) is the degree of cross resistance between the two insecticides. The inset graph is the simulation where insecticide decay does not occur and has the same axis labels.


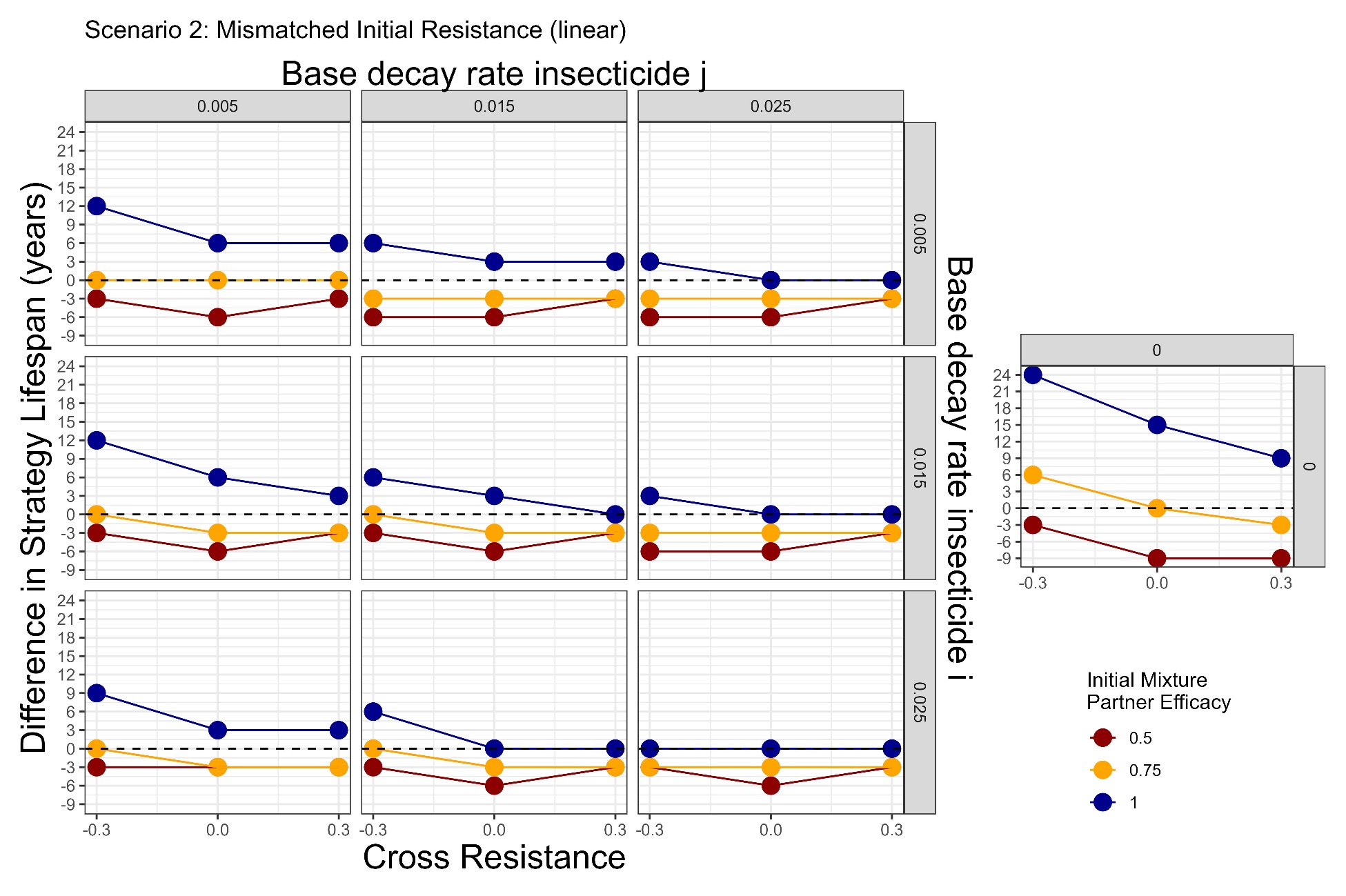


**Figure S3.3** **Impact of including insecticide decay in simulations evaluating mixtures versus monotherapy sequences with mismatched initial resistances: constant decay.** The colours indicate the deployed efficacy of each insecticide when in mixture. The horizontal dashed line indicates a difference in lifespan of zero i.e. the mixture strategy and monotherapy sequence strategy had the same strategy lifespans. Values above this line indicate the mixture strategy had a longer strategy lifespan and values below this line indicates the monotherapy sequence strategy had the longer strategy lifespan. The panels top-bottom are the base decay rate for insecticide $i$, and the panels left-right are the base decay rate for insecticide $j$. The x axis (bottom) is the degree of cross resistance between the two insecticides. The inset graph is the simulation where insecticide decay does not occur and has the same axis labels.


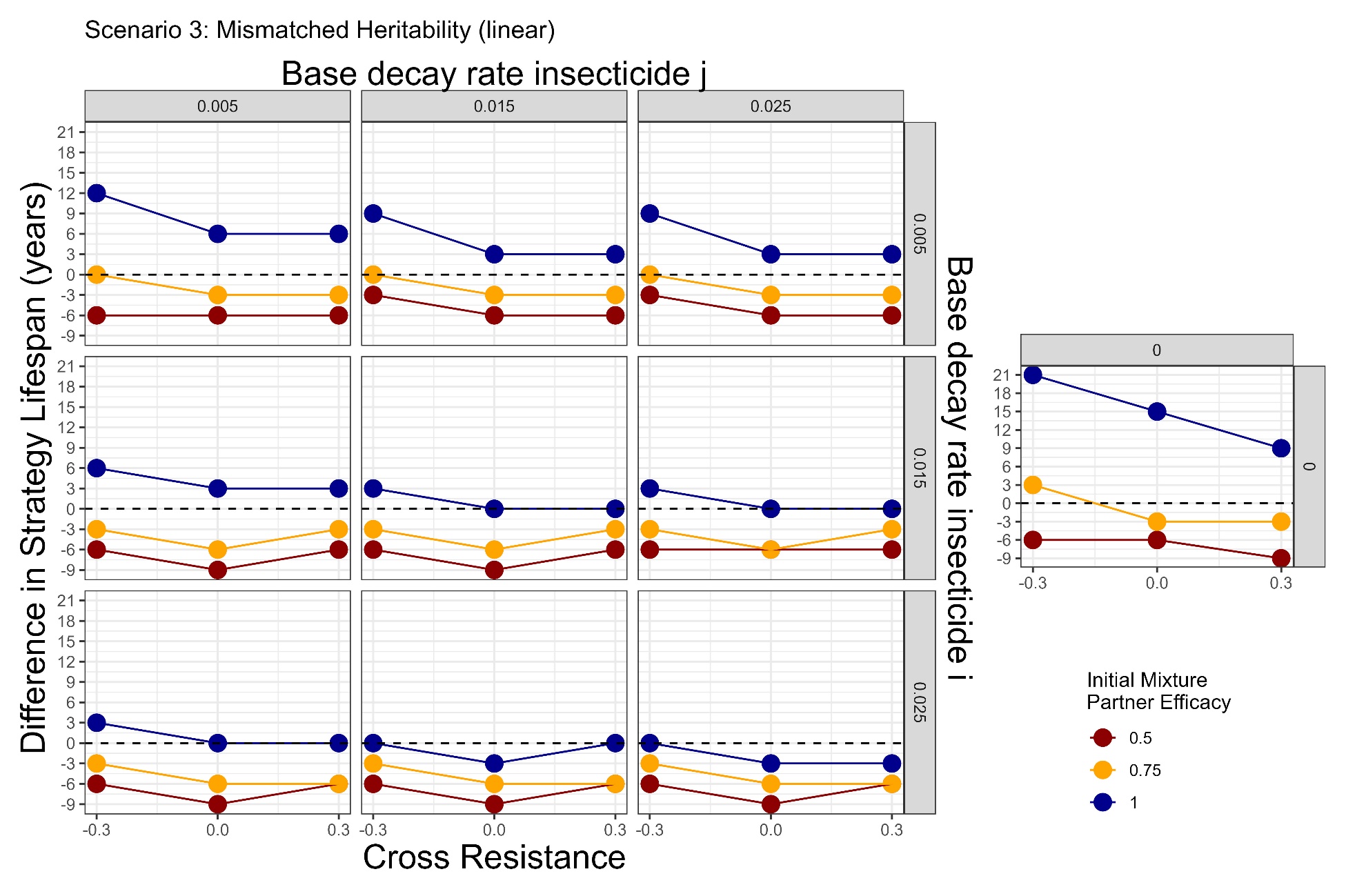


**Figure S3.4** **Scenario 3 – Impact of including insecticide decay in simulations evaluating mixtures versus monotherapy sequences with mismatched heritabilities: constant decay.** In this scenario the two insecticides had different heritabilities. The heritability for insecticide$i$ ($h_{I}^{2}$) was 0.15 and the heritability for insecticide $j$($h_{J}^{2}$) was: 0.25. The colours indicate the deployed efficacy of each insecticide when in mixture. The dashed line indicates the mixture strategy and monotherapy sequence strategy had the same strategy lifespans. Values above this line indicate the mixture strategy had a longer strategy lifespan and values below this line indicates the monotherapy sequence strategy had the longer strategy lifespan. The panels top-bottom are the base decay rate for insecticide $i$, and the panels left-right are the base decay rate for insecticide $j$. The x axis (bottom) is the degree of cross resistance between the two insecticides. The inset graph is the simulation where insecticide decay does not occur and has the same axis labels.
